# Forkhead Box Protein K1 Promotes Chronic Kidney Disease by Driving Glycolysis in Tubular Epithelial Cells

**DOI:** 10.1002/advs.202405325

**Published:** 2024-07-31

**Authors:** Lu Zhang, Maoqing Tian, Meng Zhang, Chen Li, Xiaofei Wang, Yuyu Long, Yujuan Wang, Jijia Hu, Cheng Chen, Xinghua Chen, Wei Liang, Guohua Ding, Hua Gan, Lunzhi Liu, Huiming Wang

**Affiliations:** ^1^ Department of Nephrology Renmin Hospital of Wuhan University Wuhan Hubei 430060 China; ^2^ Hubei Provincial Clinical Research Center for Kidney Disease Wuhan Hubei 430060 China; ^3^ Department of Nephrology The First Affiliated Hospital of Chongqing Medical University Chongqing 400016 China; ^4^ Hubei Provincial Clinical Medical Research Center for Nephropathy Minda Hospital of Hubei Minzu University Enshi Hubei 445000 China

**Keywords:** chronic kidney disease, FOXK1, glycolysis, phase separation, transcriptional regulation

## Abstract

Renal tubular epithelial cells (TECs) undergo an energy‐related metabolic shift from fatty acid oxidation to glycolysis during chronic kidney disease (CKD) progression. However, the mechanisms underlying this burst of glycolysis remain unclear. Herein, a new critical glycolysis regulator, the transcription factor forkhead box protein K1 (FOXK1) that is expressed in TECs during renal fibrosis and exhibits fibrogenic and metabolism‐rewiring capacities is reported. Genetic modification of the *Foxk1* locus in TECs alters glycolytic metabolism and fibrotic lesions. A surge in the expression of a set of glycolysis‐related genes following FOXK1 protein activation contributes to the energy‐related metabolic shift. Nuclear‐translocated FOXK1 forms condensate through liquid‐liquid phase separation (LLPS) to drive the transcription of target genes. Core intrinsically disordered regions within FOXK1 protein are mapped and validated. A therapeutic strategy is explored by targeting the *Foxk1* locus in a murine model of CKD by the renal subcapsular injection of a recombinant adeno‐associated virus 9 vector encoding *Foxk1*‐short hairpin RNA. In summary, the mechanism of a FOXK1‐mediated glycolytic burst in TECs, which involves the LLPS to enhance FOXK1 transcriptional activity is elucidated.

## Graphical abstract

This study identifies FOXK1 as a critical glycolysis regulator that is expressed in renal tubular epithelial cells (TECs) during renal fibrosis. FOXK1 enhances glycolytic metabolism and fibrotic lesions by forming condensates through liquid‐liquid phase separation and upregulating the transcription of glycolysis‐related genes. This study provides novel insights into the pathogenesis of chronic kidney disease and offers possible interventions.

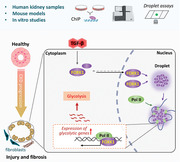

## 1 Introduction

Chronic kidney disease (CKD) has emerged as an epidemic threatening global public health. The progression of CKD, regardless of its etiology, ultimately culminates in the end stage of renal disease (ESRD) and the corollary of pathological changes associated with renal fibrosis.^[^ ^1^, ^2^ ^]^ It features myofibroblast proliferation, extracellular matrix (ECM) deposition, and loss of integrity in the glomeruli, tubules, and interstitial capillaries.^[^ ^3^, ^4^ ^]^ The pathogenetic pathways underlying renal fibrosis involve many cell types and mediators that form a complex interactive network. Renal tubular epithelial cells (TECs) act as the core component in the regulatory network facilitating renal fibrosis. It has been revealed that TECs are not only the victims of renal injury but also the powerful drivers in developing fibrosis.^[^ ^5^ ^]^ TECs undergo a substantial transition in phenotype and function upon the stimulus of repeated or prolonged insults. The profound alterations in many aspects, for instance, morphology, structure, composition, reabsorption, and secretion, have been observed in injured TECs as the maladaptive response accompanying fibrotic lesion.^[^ ^5^, ^6^ ^]^ Apart from that, the switch of cellular metabolism of TECs occurred as a critical process that not only signifies but also underpins the fibrotic lesion in kidney tissue.^[^ ^7^ ^]^

Renal tubules constitute the major components of renal parenchyma in terms of tissue volume and cell numbers.^[^ ^7^ ^]^ Besides providing integrity to renal tubular architecture, TECs play a pivotal role in maintaining body homeostasis by carrying out physiological transport functions, active reabsorption of nutrients and electrolytes in the tubules, and active secretion of unneeded compounds in an energy‐consuming manner.^[^ ^8^ ^]^ Renal tubules, particularly the proximal segments, have a large energy demand in the physiological state and adopt the preferred pattern of energy supply through fatty acid oxidation (FAO), with gluconeogenesis as a supplemental conduit.^[^ ^9^ ^]^ However, cellular energy metabolism is altered when insufficient oxygen supply or other insults are present.^[^ ^10^ ^]^ Previous studies have shown that mitochondrial dysfunction^[^ ^11^, ^12^, ^13^, ^14^ ^]^ and FAO downregulation.^[^ ^11^, ^15^, ^16^, ^17^ ^]^ occur during TECs injury. In addition, enhanced glycolysis was observed in the fibrotic kidneys of CKD patients.^[^ ^18^ ^]^ However, the direct evidence supporting the robust glycolysis in TECs in the context of fibrotic kidneys is still lacking. Moreover, the core regulators and mechanisms involved in triggering glycolysis in TECs during CKD development need to be elucidated, considering that FAO and glycolysis are divergent metabolic pathways, although they are cross‐linked.

FOXK1 is a transcription factor belonging to the FOX family that shares multiple functions. Data from other studies have indicated the essential role of FOX family members in organ fibrosis.^[^ ^19^, ^20^, ^21^, ^22^, ^23^, ^24^ ^]^ As a member of the FOX family, FOXK1 possesses fundamental roles in regulating a few cellular processes, including autophagy and cell proliferation.^[^ ^25^, ^26^ ^]^ What is noteworthy is the reports that FOXK1 mediated aerobic glycolysis in adipocyte cells via regulating glycolysis‐related enzymes, hexokinase‐II (HK2) and pyruvate kinase M2 (PKM2).^[^ ^27^ ^]^ The role of FOXK1 in CKD and TECs glycolysis, however, remain largely unexplored. In this study, we aimed to investigate the role of FOXK1 in initiating glycolysis within TECs and renal fibrosis progression.

## 2 Results

### 2.1 FOXK1 Expression was Upregulated in TECs During CKD Progression

**Figure 1:**
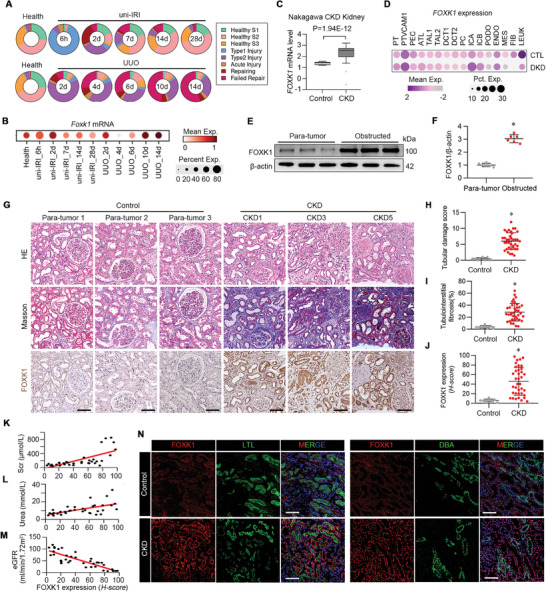
FOXK1 is significantly upregulated in fibrotic kidney featuring proximal TEC‐specific distribution. A) Pie diagrams displaying the proportional abundance of each cell cluster in each disease condition. B) *Foxk1* gene expression analysis of a scRNA‐seq dataset (GSE190887) sourced from mice kidneys challenged with uni‐IRI or UUO at different time points. C) Relative mRNA level of *FOXK1*. FOXK1 expression in CKD kidney tissues from Nephroseq database (“Nakagawa CKD kidney” dataset, median‐centered log2). Unpaired t‐test. D) Single‐cell analysis of *FOXK1* expression in kidney tissues from Diabetic Kidney Disease (DKD) and in para‐tumor kidney tissues from renal cancer patients as normal control. The samples were retrieved from the GSE151302, GSE131882 databases. PCT, proximal convoluted tubule; PTVCAM1, VCAM1(+) proximal tubule; PEC, parietal epithelial cells; ATL, ascending thin limb; TAL1, CLDN16(‐) thick ascending limb; TAL2, CLDN16(+) thick ascending limb; DCT1, early distal convoluted tubule; DCT2, late distal convoluted tubule; PC, principal cells; ICA, type A intercalated cells; ICB, type B intercalated cells; PODO, podocytes; ENDO, endothelial cells; FIB, fibroblasts; MES, mesangial cell; LEUK, leukocytes. E, F) Western blotting and statistical analysis of the FOXK1 expression in kidney tissues obtained from patients with obstructive nephropathy or para‐tumor tissue as control (*n* = 6 for each group). G) H&E, Masson, and immunohistochemistry staining in human kidney specimens from CKD patients or control subjects (para‐tumor group). Scale bar = 100 µm. H–J) Quantification of tubular damage score (H), and tubulointerstitial fibrosis percentage (I), FOXK1 protein expression (J) based on H&E, Masson, and immunohistochemistry staining in (G). K–M) Correlation between kidney FOXK1 expression and serum creatinine (Scr) (K), and blood urea nitrogen (L), and estimated glomerular filtration rate (eGFR) (M) for patients with CKD. N Immunofluorescence staining of FOXK1 (red) and LTL (green) or DBA (green) in human kidneys, DAPI (blue). Scale bar = 100 µm.

To determine the role of FOXK1 in CKD progression, we first analyzed the single‐cell RNA sequencing dataset, GSE190887,^[^ ^28^ ^]^ related to mouse unilateral ureteral obstruction (UUO) and unilateral ischemia‐reperfusion injury (uni‐IRI) that was deposited in the Gene Expression Omnibus (GEO) database. This indicates that the composition of the TECs subpopulation, corresponding to different cell conditions or fates, is dynamically altered in accordance with disease progression, as either AKI or CKD. In the AKI disease model of uni‐IRI, an injury cluster surges immediately at the early stage followed by an increase in repairing and failed repaired (FR) clusters, which culminate at day 2 and day 7 respectively, vanish gradually at a later stage, and finally replaced by a healthy cluster, signaling a recovery from AKI (**Figure** **1**A). In the CKD model of UUO, the TECs clusters exhibit a distinct dynamic pattern, characterized by an FR cluster that grows continuously along with the development of CKD and spreads through the whole TECs population in the late stage, indicating renal fibrosis (Figure 1A). We further depicted the expression profile of the *Foxk1* by disease stage. We observed that *Foxk1* was weakly expressed in healthy TECs, and was slightly downregulated at 6 h after uni‐IRI but rebounded markedly to high expression levels at day 2. Its expression gradually decreased during the repair phase after uni‐IRI at day 7, followed by a stable restoration to approximately normal level until day 28. In the CKD model, *Foxk1* expression was slightly elevated on day 2 after UUO and was reduced to a low level from day 4 to day 6, followed by a marked elevation from day 10 to day 14 (Figure 1B). It suggested that the dynamic trajectory of *Foxk1* expression is in alignment with the cell fate transition and the disease development. On top of that, analysis of the “Nakagawa CKD kidney” dataset from the renal transcriptomics database Nephroseq indicated that *FOXK1* mRNA was significantly upregulated in kidney tissues from patients with CKD in comparison with the normal controls (*P *= 1.94E‐12) (Figure 1C). Meanwhile, the analysis of single‐cell sequencing datasets GSE151302 and GSE131882,^[^ ^29^, ^30^ ^]^ generated from diseased and normal human kidney tissues, yielded comparable results (Figure 1D). Evermore, we examined FOXK1 expression in kidney tissues of patients with obstructive nephropathy, or in the para‐tumor kidney tissues of renal cancer patients undergoing nephrectomy. As expected, the protein level of FOXK1 was significantly upregulated in the kidneys of obstructive nephropathy compared with para‐tumor (Figure 1E,F). Then, we analyzed micro‐dissected human kidney samples collected from patients with CKD (*n* = 37) and normal tissues adjacent to the tumor (Non renal fibrosis group, *n* = 8). The clinical demographics of these subjects are provided in Table S1–S2 (Supporting Information). Patients with CKD showed tubular injury and collagen deposition as captured by Hematoxylin‐Eosin (H&E) and Masson staining (Figure 1G–I). Immunohistochemistry (IHC) staining showed that FOXK1 expression was upregulated in patients with CKD, and the expression intensity was increased along with the CKD stages (Figure 1G,J). In addition, FOXK1 expression level showed a positive correlation with the level of serum creatinine (Figure 1K), and the blood urea nitrogen (Figure 1L); and with a negative correlation with the estimated glomerular filtration rate (eGFR) (Figure 1M). The IHC staining revealed that the induced FOXK1 was mainly distributed in tubules, and more precisely in the proximal renal tubules as detected by immunofluorescence staining, which showed the co‐localization of FOXK1 with the proximal tubule marker LTL (Lotus tetragonolobus lectin) but not with the collecting duct marker DBA (Dolichos biflorus agglutinin) (Figure 1N).

### 2.2 TEC‐Specific *Foxk1* Knockout Attenuated Renal Fibrosis in Mouse Models

**Figure 2:**
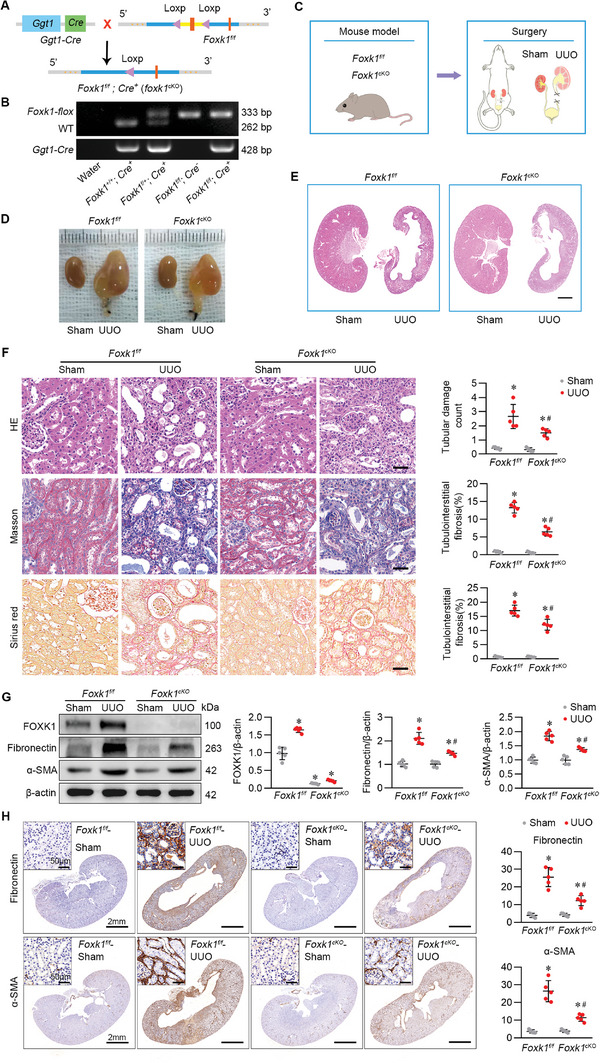
Tubular‐specific *Foxk1*‐deletion ameliorated kidney fibrosis in UUO model. A) Schematic diagram of the generation and identification of TEC‐specific *Foxk1* knockout mouse (*Foxk1 ^cKO^*) and the control mouse with regular FOXK1 expression (*Foxk1 ^flox/flox^*). B) Genotyping of *Foxk1* and *Cre* mice. PCR assay of mice from the indicated groups. C) Schematic diagram of generation of the UUO mouse model. Seven days after UUO, mice were sacrificed for kidney collection. D) Gross appearance of kidneys from the indicated groups. E) Photomicrographs exhibiting the Hematoxylin and eosin (H&E) staining of kidney sections from the indicated groups. Scale Bar = 2 mm. F) H&E, Masson staining, and Sirius red staining were applied to examine the tubular lesion and interstitial fibrosis in the renal tissue section from the indicated groups. Scale Bar = 50 µm. *n* = 5 mice per group. G) Western blotting of the protein expression of the related molecules in kidney tissues from the indicated group. *n* = 5 mice per group. Quantitative data are expressed as the mean ± S.E.M. *p < 0.05, compared to the sham group; *^#^p *< 0.05, compared with *Foxk1 ^flox/flox^*‐UUO mice. H) IHC staining of the protein expression of the related molecules in kidney tissues from the indicated group. *n* = 5 mice per group. Quantitative data are expressed as the mean ± S.E.M. *p < 0.05, compared to the sham group; *^#^p *< 0.05, compared with *Foxk1 ^flox/flox^*‐UUO mice.

The above results indicated that FOXK1 induction in the kidney is in a fibrosis‐associated and tubule‐specific manner. We further investigated the role of FOXK1 in kidney fibrosis development. To this end, conditional tubular‐specific *Foxk1*‐deficient (*Foxk1* ^*cKO*^) mice (Figure S1, Supporting Information) were established by crossing mice with the genetic background of a conditional allele knockout of *Foxk1 (Foxk1 ^flox/flox^)* with that of *Ggt1‐Cre* mice (**Figure** **2**A,B; Figure S2A, Supporting Information). *Foxk1* ^*cKO*^ mice and the control *Foxk1 ^flox/flox^* mice underwent UUO surgery (Figure 2C) and were sacrificed at the indicated time. The kidney cortex of *Foxk1 ^flox/flox^* mice that had undergone UUO was thinner and paler in appearance compared with those of corresponding *Foxk1* ^*cKO*^ mice (Figure 2D,E). UUO treatment caused fibrotic lesions in the kidney featuring tubular atrophy dilatation, and interstitial collagen deposition, which was significantly mitigated in *Foxk1* ^*cKO*^ mice in comparison with *Foxk1 ^flox/flox^* mice (Figure 2F). Next, we detected the protein levels of the classic fibrotic genes and found that Fibronectin (FN) and Alpha‐smooth muscle actin (α‐SMA) were upregulated in the UUO kidney of *Foxk1 ^flox/flox^* mice, and this induction was largely reduced in *Foxk1 ^cKO^* mice. The indicated protein level variation in the kidney tissues was consistent with the mRNA level (Figure 2G,H). On top of the studies based on the UUO disease model, similar experiments were carried out parallelly in IRI mice, another progressive CKD mouse model. In the IRI‐induced renal fibrosis model, the gross renal morphology, results of histochemistry staining, and fibrotic marker expression levels were recapitulated and consistent with that in the UUO model (Figure S2, Supporting Information), thus corroborating the pathogenic role of FOXK1 in renal fibrosis development in mice.

### 2.3 *FOXK1* Knockdown Attenuated TGF‐β1 Elicited Pro‐Fibrotic Effects in Cultured Tubular Cells

**Figure 3:**
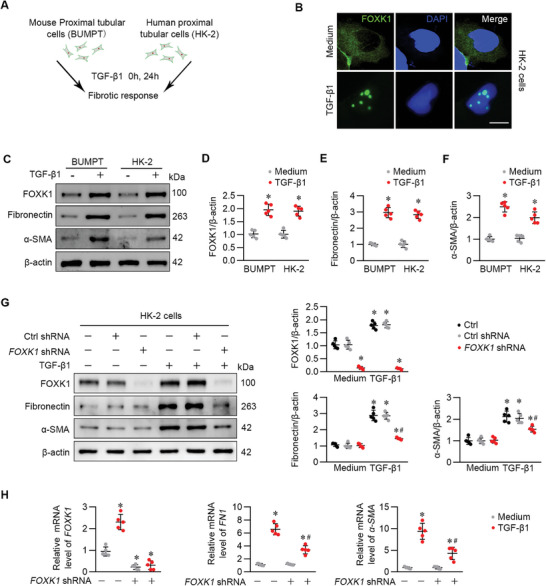
FOXK1 exerted pro‐fibrotic effects on TECs under TGF‐β1 stimulation. A) Schematic diagram of the in vitro experiments on renal tubular cells. Mouse proximal tubular cells (BUMPT) and human proximal tubular cells (HK‐2) were treated with 10 ng mL^−1^ TGF‐β1 for the indicated times. B) Immunofluorescence analysis was performed to detect the expression and distribution of FOXK1 in HK‐2 cells from the indicated groups. Scale Bar = 20 µm. C–F) Western blotting and statistical analysis of the protein expression of the related molecules in BUMPT cells or HK‐2 cells from the indicated group. Quantitative data are expressed as the mean ± S.E.M. *p < 0.05, compared to the control group. G) Western blotting and statistical analysis of the protein expression of the related molecules in HK‐2 cells from the indicated group. Quantitative data are expressed as the mean ± S.E.M. *p < 0.05, compared to the control group. *^#^p *< 0.05, compared to TGF‐β1‐stimuli group. H) Relative mRNA levels of *FOXK1, FN1*, and *α‐SMA* in HK‐2 cells after transfected with *FOXK1* shRNA or control shRNA, followed by TGF‐β1 treatment. Quantitative data are expressed as the mean ± S.E.M. *p < 0.05, compared to the control group. *^#^p *< 0.05, compared to the TGF‐β1‐stimuli group. Representative images are shown from at least 3 independent experiments. Ctrl, control; TGF‐ β1, transforming growth factor β−1; FN1, Fibronectin; α‐SMA, alpha‐smooth muscle actin.

The above findings from in vivo experiments revealed that FOXK1 expression in tubules, predominantly distributed at the proximal segment of the nephron, aggravated the progression of renal fibrosis. We next examined the expression of FOXK1 and its role in cultured tubular cells under fibrogenic stimulation (**Figure** **3**A). Cultured murine proximal tubular cells (BUMPT cells) and those of human origin (HK‐2 cells) were treated with TGF‐β1. Immunofluorescence staining showed that TGF‐β1‐induced FOXK1 was mainly present within the nucleus in HK‐2 cells (Figure 3B). FOXK1 and fibrotic markers FN and α‐SMA, either in protein or in mRNA, were markedly upregulated remarkably under TGF‐β1 stimulation (Figure 3C–F). In tubular cells with *FOXK1* knockdown by short hairpin RNA (shRNA) transfection, the upregulation of FN and α‐SMA in response to TGF‐β1 stimulation were significantly suppressed when compared with cells receiving control shRNA transfection (Figure 3G,H). These results from in vitro experiments suggested that fibrogenic factor TGF‐β1 induces FOXK1 expression and nuclear translocation, which is conducive to the pro‐fibrotic effects on TECs.

### 2.4 TGF‐β1 Treatment Increased Genome‐Wide FOXK1 Occupancy

**Figure 4:**
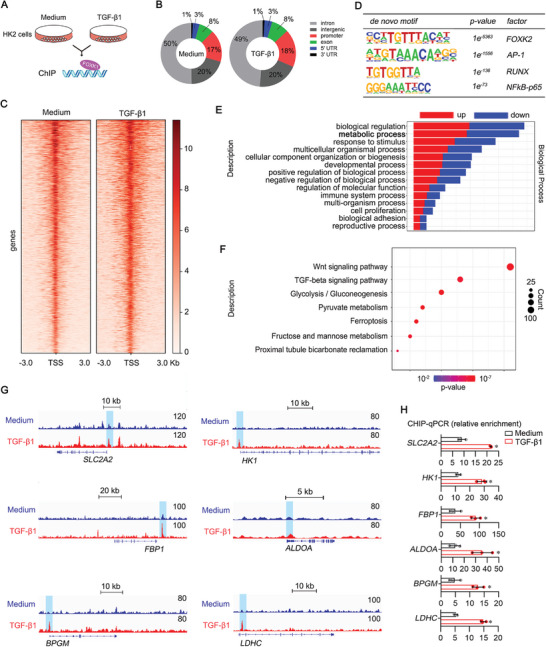
FOXK1 regulated glycolytic gene transcription. A) Schematic diagram of the ChIP‐seq analysis on HK‐2 cells under indicated treatment. B) Genomic distribution of FOXK1 in HK‐2 cells treated with TGF‐β1 or not for 24 h. C Heatmaps of ChIP‐seq signals (TSS ± 3 kb) for FOXK1 from the indicated groups. D) Motif analysis of genome‐wide FOXK1 binding sites, assessed by the HOMER motif analysis algorithm. E) The Gene Ontology analysis for the FOXK1 target genes induced by TGF‐β1. F) Kyoto Encyclopedia of Genes and Genomes (KEGG) analysis for the FOXK1 target genes induced by TGF‐β1. G) Visualization of ChIP‐seq data for FOXK1 target genes (*SLC2A2, HK1, FBP1, ALDOA, BPGM, LDHC*) in the genomic region. H) ChIP‐qPCR was performed to verify FOXK1 binding to the promoter of glycolysis‐related genes (*SLC2A2, HK1, FBP1, ALDOA, BPGM, LDHC*) in response to TGF‐β1 treatment in HK‐2 cells (*n* = 3). *p < 0.05, compared to the control group.

FOXK1 is a transcription factor involved in glucose metabolism, cell proliferation, and autophagy, and the functions are exercised mainly through transcriptional regulation of its target genes.^[^ ^26^, ^27^, ^31^, ^32^ ^]^ To explore the transcriptional regulatory mechanisms underlying the roles of FOXK1 in renal fibrosis, we performed chromatin immunoprecipitation‐sequencing (ChIP‐seq) to analyze the genome‐wide distribution of FOXK1 in HK‐2 cells (**Figure** **4**A). We found that TGF‐β1 treatment substantially boosted FOXK1 occupancy on chromatin, with peak binding sites up from 1718 to 2168 (Figure 4B). As depicted in the heat maps in Figure 4C, 24 h of TGF‐β1 stimulation also increased FOXK1 signal intensity over the genic regions. Motif analysis identified several transcription factors binding sites near the FOXK1 occupied area, including FOXK2, AP1, RUNX, and NF‐κB in TGF‐β1‐treated or untreated HK‐2 cells (Figure 4D). The Gene Ontology analysis of the peak‐associated genes revealed that the highly enriched FOXK‐bound genes were related to the metabolic process (Figure 4E). Kyoto Encyclopedia of Genes and Genomes (KEGG) showed that genes associated with FOXK1 were enriched for the glycolysis/gluconeogenesis pathway (Figure 4F; Table S3, Supporting Information). Gene tracks in Figure 4G indicated FOXK1 ChIP‐seq peaks for glycolysis‐related genes (*SLC2A2, HK1, FBP1, ALDOA, BPGM, LDHC*); TGF‐β1 stimulation caused an increase in FOXK1 binding. We further confirmed that FOXK1 binds to the transcription start sites (TSS) of these six genes by ChIP‐qPCR assay (Figure 4H; Figure S3, Supporting Information). These results based on ChIP‐seq and quantitative reverse transcriptase‐polymerase chain reaction (qRT‐PCR) data identified the FOXK1 target genes related to glycolysis switch in TECs in response to fibrogenic stimulation via TGF‐β1.

### 2.5 FOXK1 Boosted TECs Glycolysis and Impacted Mitochondrial Function

**Figure 5:**
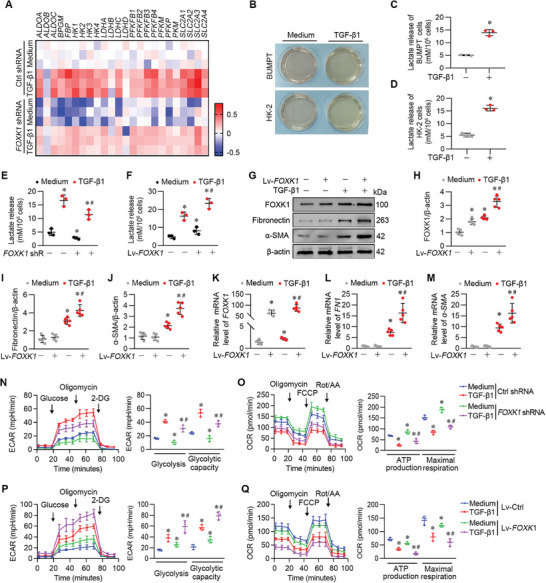
FOXK1 triggered glycolysis through transcriptional regulation of glycolytic‐related genes. A) Heat map showing the expression profile of glycolysis‐related gene sets in HK‐2 cells transfected with *FOXK1* shRNA or control shRNA in the presence of TGF‐β1 or control medium. B) Culture medium color of the BUMPT or HK‐2 cells from the indicated groups. C–F) The concentration of lactate was detected by ELISA kit from the indicated groups. In some conditions, HK‐2 cells were transduced with lentivirus vectors carrying *FOXK1* encoding or scrambled plasmid to generate *FOXK1* overexpression (Lv‐*FOXK1*) or controlled (Lv‐ctrl) cells. Data are representative of 3 independent experiments. *p < 0.05, compared to the control group. *^#^p *< 0.05, compared to the TGF‐β1‐stimuli group. G–J) Western blotting and statistical analysis of the protein expression of the related molecules in HK‐2 cells from the indicated group. *p < 0.05, compared to the control group. *^#^p *< 0.05, compared to the TGF‐β1‐stimuli group. K–M) qRT‐PCR analysis of the expression of the related molecules in HK‐2 cells from the indicated group. *p < 0.05, compared to the control group. *^#^p *< 0.05, compared to the TGF‐β1‐stimuli group. N) Extracellular acidification rate (ECAR) in cultured HK‐2 cells knocked down for *FOXK1* in the presence of TGF‐β1 or control medium. Statistical analyses of glycolysis and glycolytic capacity in ECAR. 2‐DG, 2‐deoxy‐D‐glucose (*n* = 3 per group). *p < 0.05, compared to the control group. *^#^p *< 0.05, compared to the TGF‐β1‐stimuli group. O) Oxygen consumption rate (OCR) in cultured HK‐2 cells knocked down for *FOXK1* in the presence of TGF‐β1 or control medium. Statistical analyses of ATP production and maximal respiration in OCR (*n* = 3 per group). *p < 0.05, compared to the control group. *^#^p *< 0.05, compared to the TGF‐β1‐stimuli group. P) ECAR in cultured HK‐2 cells overexpressing for *FOXK1* in the presence of TGF‐β1 or control medium. Statistical analyses of glycolysis and glycolytic capacity in ECAR. 2‐DG, 2‐deoxy‐D‐glucose (*n* = 3 per group). *p < 0.05, compared to the control group. *^#^p *< 0.05, compared to the TGF‐β1‐stimuli group. Q) Oxygen consumption rate (OCR) in cultured HK‐2 cells overexpressing for FOXK1 in the presence of TGF‐β1 or control medium. Statistical analyses of ATP production and maximal respiration in OCR (*n* = 3 per group). *p < 0.05, compared to the control group. *^#^p *< 0.05, compared to the TGF‐β1‐stimuli group.

We then evaluated the role of FOXK1 in regulating glycolysis in cultured TECs. In this study, based on related articles^[^ ^27^, ^33^ ^]^ and the Molecular Signatures Database (MSigDB), 24 glycolysis‐related genes were curated (**Figure** **5**A). The heat map of differential expression genes (DEG) revealed that glycolysis‐related genes were perturbed by the FOXK1 modification and TGF‐β1 treatment. In addition to *SLC2A2, HK1, FBP1, ALDOA, BPGM*, and *LDHC* genes, an array of glycolysis‐related genes including *PKM, HK2, LDHA*, and et al, were found subjected to FOXK1 modification (Figure 5A). As the principal pro‐fibrotic factor in renal fibrosis, TGF‐β1 triggers a metabolism reprogramming with a shift toward glycolysis in fibroblast,^[^ ^34^, ^35^ ^]^ which seems to be the same case for TGF‐β1 in TECs. We observed that the culture medium of BUMPT cells and HK‐2 cells exhibited a markedly acidic appearance (Figure 5B) and higher lactate concentrations (Figure 5C,D) in the presence of TGF‐β1. Knocked down of FOXK1 significantly downregulated the protein levels of a panel of glycolysis‐related molecules, including SLC2A2, HK1, FBP1, ALDOA, BPGM, LDHC, PKM2, HK2, LDHA (Figure S4A,B, Supporting Information), and resulted in less lactate production (Figure 5E) in HK‐2 cells. In contrast, elevated expression of FOXK1 in HK‐2 cells resulted in more lactate production (Figure 5F), upregulation of the fibrotic markers of Fibronectin and α‐SMA at both the protein (Figure 5G–J), and mRNA levels (Figure 5K,M) upon TGF‐β1 stimulation, as well as the rise of the glycolysis‐related genes (Figure S4C,D Supporting Information). We also measured the lactate level and the expression level of the glycolysis‐related genes in fibrotic mouse kidneys. As expected, *Foxk1* deficiency led to a significant reduction of lactate production (Figure S5A,B, Supporting Information) and the concerned molecules expression in both mRNA levels (Figure S5C,D, Supporting Information) and protein levels (Figure S6, Supporting Information) in isolated renal tubules from *Foxk1* ^*cKO*^ mice that underwent UUO or IRI surgery.

Besides, we measured the extracellular acidification rate (ECAR) and oxygen consumption rate (OCR) of HK‐2 cells following TGF‐β1 stimulation. Our data showed that TGF‐β1 stimulation elevated the ECAR and lowered the OCR in HK‐2 cells, and these effects were dramatically dampened when FOXK1 was silenced (Figure 5N,O) and were heightened when the FOXK1 regained expression (Figure 5P,Q). Taken together, these results demonstrated that FOXK1 is critical in the energic shift to glycolysis in TECs under diseased conditions.

### 2.6 FOXK1 Exerted Transcriptional Activities Depending on Liquid‐Liquid Phase Separation (LLPS)

**Figure 6:**
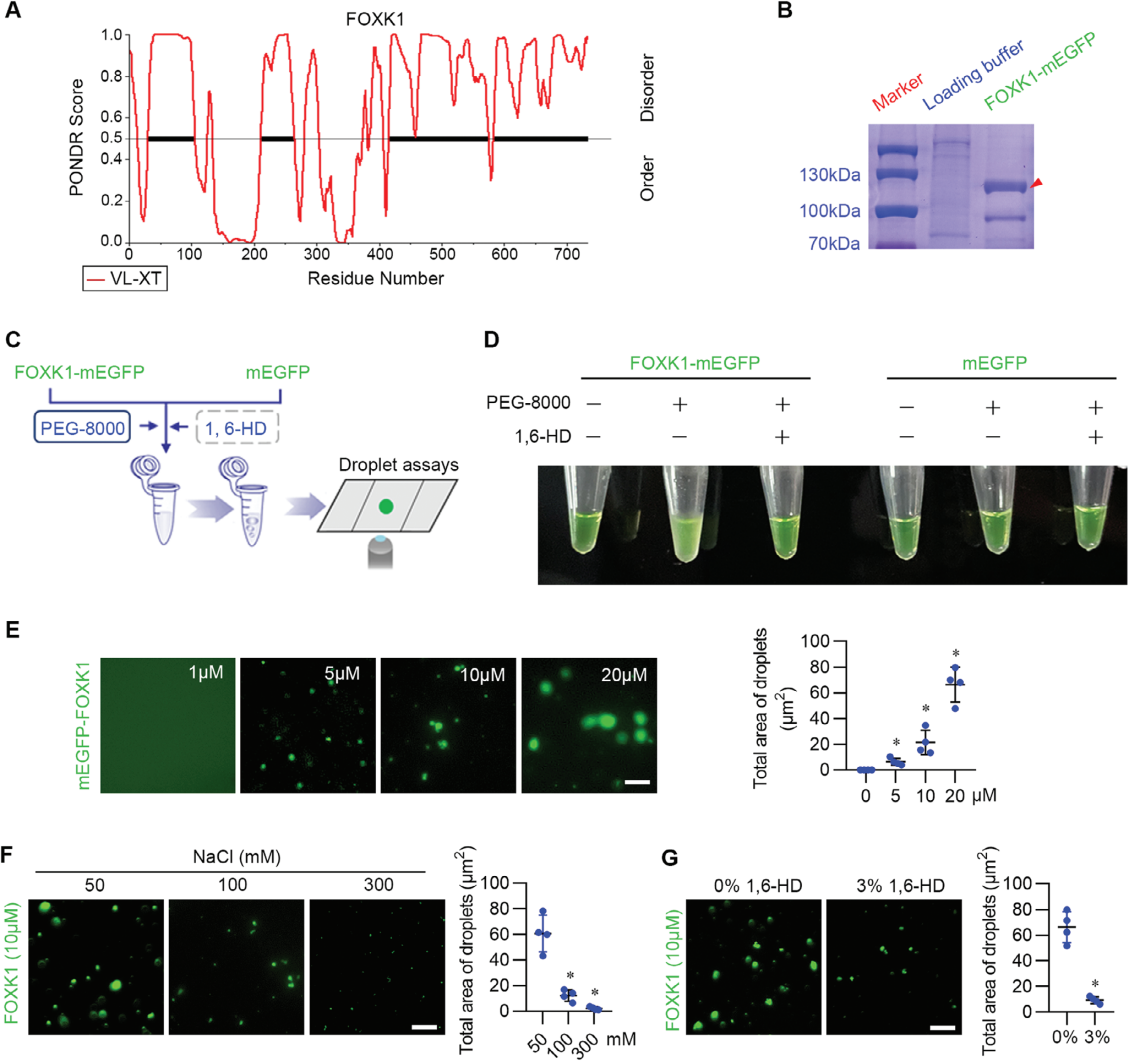
FOXK1 protein possesses the capacity to form liquid droplets. A) Graph of intrinsically disordered regions of FOXK1 as calculated by the VL‐XT algorithm (http://pondr.com/). The x‐axis indicates the position of the amino acid, and the y‐axis shows the score of PONDR VL‐XT. For PONDR prediction, a score of more than 0.5 indicates a high degree of disorder. Heavy bars indicate IDRs. B) Recombinant FOXK1‐mEGFP fusion protein resolved on an 8% SDS–PAGE gel and stained with Coomassie brilliant blue. C) Schematic illustration of droplet formation assay in vitro. D) Visualization of solution turbidity associated with droplet formation in vitro. Tubes containing FOXK1‐mEGFP or vector‐mEGFP in the presence (+) or absence (−) of PEG‐8000 or 1,6‐HD are shown. E) Representative fluorescence images of droplet formation at different concentrations of FOXK1‐mEGFP fusion protein or vector‐mEGFP were added to the droplet formation buffer to final concentrations as indicated. Quantification of the size and number of droplets are shown. Each dot represents a droplet. Data are mean ± S.E.M. Scale Bar = 5 µm. F) Representative images of droplet formation at different concentrations of NaCl solution as indicated. Representative images of dissolved droplets. FOXK1‐mEGFP in the 0% or 3% of 1,6‐HD are shown. Quantification of the area of droplets is shown. Each dot represents a droplet. Data are mean ± S.E.M. Scale Bar = 5 µm. G) Representative images of dissolved droplets. FOXK1‐mEGFP in the 0% or 3% of 1,6‐HD are shown. Quantification of the area of droplets is shown. Each dot represents a droplet. Data are expressed in mean ± S.E.M. Scale Bar = 5 µm. Two‐tailed unpaired Student's t‐test was used for statistical analysis. *p < 0.05, compared to the control group.

Intracellular biomacromolecules, such as proteins and nucleic acids, self‐assembled into liquid‐like condensates through a physicochemical mechanism of LLPS,^[^ ^36^, ^37^ ^]^ which provides essentially membrane‐less compartments with concentrated and segregated cellular components to fulfill a range of distinct physiological functions including transcriptional activities in living cells.^[^ ^38^, ^39^ ^]^ Based on sequence analysis and prediction, we found that FOXK1 protein contains four intrinsically disordered regions (IDRs) that are vital for forming liquid condensates (**Figure** **6**A), indicating the nature of FOXK1 to form condensates. A recombinant FOXK1‐mEGFP (monomeric enhanced green fluorescent protein) fusion protein was then produced and purified (Figure 6B,C). The addition of FOXK1‐mEGFP to the buffers containing 10% polyethylene glycol‐molecular weight 8000 (PEG‐8000) buffer resulted in the solution becoming opaque, while the solution remained clear when adding mEGFP proteins. The 1,6‐hexanediol (1,6‐HD), an aliphatic alcohol, is widely used for dissolving LLPS droplets. As expected, adding 1,6‐HD made the solution clear (Figure 6D). Droplet formation assay revealed that FOXK1‐mEGFP formed phase‐separated spherical droplets in a concentration‐dependent manner, while mEGFP remained diffuse in all conditions tested (Figure 6E). The number of droplets and the total area of droplets of FOXK1‐mEGFP were negatively dependent on NaCl or 3% 1,6‐hexanediol concentration (Figure 6F,G).

**Figure 7:**
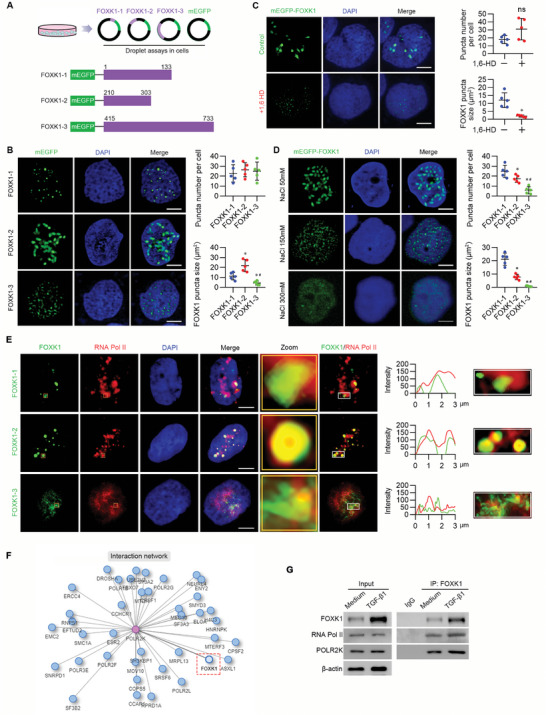
FOXK1 IDRs interact with RNA PoI II in liquid droplets inside the living cells. A) Schematic diagram of plasmid constructions of FOXK1 intrinsically disordered domains. Schematic showing the plasmids design and construction of IDRs of FOXK1. B) Representative images of nuclear puncta in HK‐2 cells transfected with different IDRs of FOXK1 fused to mEGFP: Lv‐FOXK1‐1‐mEGFP, Lv‐FOXK1‐2‐mEGFP, Lv‐FOXK1‐3‐mEGFP lentivirus vectors, Nuclei are stained with DAPI (blue). For each group, 30 cells (*n*  =  30) were quantified for the average number and size of puncta using ImageJ. Scale bar = 5 µm. Data are representative of 4 to 5 experiments. Data shown as the mean ± S.E.M.; two‐tailed unpaired Student's t‐test. C) Immunofluorescence imaging of mEGFP‐FOXK1‐2 in HK‐2 cells with or without 3% hexanediol treatment for 15 s. Quantification of the average number and size of puncta are shown on the right panel. Scale bar = 5 µm. D) Representative images of mEGFP‐FOXK1‐2 in HK‐2 cells at different concentrations of NaCl solution as indicated. Quantification of the average number and size of puncta are shown on the right panel. Data shown as the mean ± S.E.M. *P* value was determined by unpaired two‐tailed Student's t‐test. Scale Bar = 5 µm. *p < 0.05, compared to the control group. E) Representative image of HK‐2 cells transfected with mEGFP‐tagged FOXK1‐1, FOXK1‐2, FOXK1‐3 plasmids and stained with RNA Pol II (red) antibody and costained with DAPI (blue). Yellow frames show a higher magnification of the indicated areas. The images were captured using confocal microscopy. The related intensity profiles of FOXK1 with RNA PoI II were analyzed (white frames) by Zen Blue version 3.1. Scale bar = 5 µm. F) The interaction network of FOXK1 by Integrated Interactions Database (http://iid.ophid.utoronto.ca/SearchPPIs/protein/). G) Immunoprecipitation assay to detect the interaction between endogenous FOXK1 and RNA Pol II or POLR2K unit in HK‐2 cells with TGF‐β1 administration for 24 h.

To determine the contributions of individual FOXK1 IDRs to phase separation, we generated three peptides using mEGFP‐tagged plasmids: peptide containing the first IDR (FOXK1^1‐133^), peptide containing the second IDRs (FOXK1^210‐303^), and peptide containing the third and fourth IDRs (FOXK1^415‐733^) (**Figure** **7**A). Droplet formation assay revealed that FOXK1^210‐303^‐mEGFP exhibited larger and more liquid droplets than FOXK1^1‐133^‐mEGFP and FOXK1^415‐733^‐mEGFP at the indicated concentration in the nucleus of HK‐2 cells (Figure 7B). The pre‐formed droplets of FOXK1^210‐303^‐mEGFP decreased in size and number with 1,6‐hexanediol treatment or NaCl treatment (Figure 7C,D). These results indicated that FOXK1 droplets are highly dynamic and that FOXK1 can form condensates in the nucleus; the IDRs (210‐303 aa) of FOXK1 had relatively better droplet formation ability than IDRs (1‐133 aa and 415–733 aa).

### 2.7 FOXK1 Condensates Encompass Essential Transcription Proteins for Active Gene Transcription

Previous studies have found that phase separation is involved in the transcriptional elongation activity of RNA polymerase II (RNA Pol II) at the promoter regions.^[^ ^40^, ^41^ ^]^ To determine whether FOXK1 condensates are involved in the assembly of RNA Pol II transcription regulatory complexes, we first assessed the colocalization of RNA Pol II with the IDRs of FOXK1. As shown in Figure 7E, compared with the other two IDRs, FOXK1^210‐303^‐mEGFP was colocalized with RNA Pol II and formed large condensates. We further analyzed the interaction network of FOXK1 using the Integrated Interactions Database (http://iid.ophid.utoronto.ca/SearchPPIs/protein/) to find the subunit of RNA Pol II responsible for the interaction with FOXK1. As shown in Figure 7F, FOXK1 interacted with POLR2K (RNA Pol II, subunit K), one of the subunits of RNA Pol II. We performed a co‐immunoprecipitation assay to validate the predicted interaction, and the results showed that FOXK1 interacted with RNA Pol II and POLR2K in HK‐2 cells (Figure 7G). Given the potential of FOXK1 to regulate the transcription of glycolytic genes, we examined the effects of IDR peptides on FOXK1‐targeted glycolytic gene expression by using full‐length FOXK1‐mEGFP or the IDR peptides expressing cells. As shown in Figure S7 (Supporting Information), the FOXK1‐mEGFP increased the mRNA levels of FOXK1 targeted glycolysis‐related genes (*Slc2a2, Hk1, Fbp1, Aldoa, Bpgm, Ldhc*), indicating that the IDRs are critical for driving FOXK1 transcriptional function.

### 2.8 Renal Subcapsular (SC) Administration of AAV9‐sh*Foxk1* Significantly Attenuated Kidney Fibrotic Lesion in UUO and IRI Murine Model

**Figure 8:**
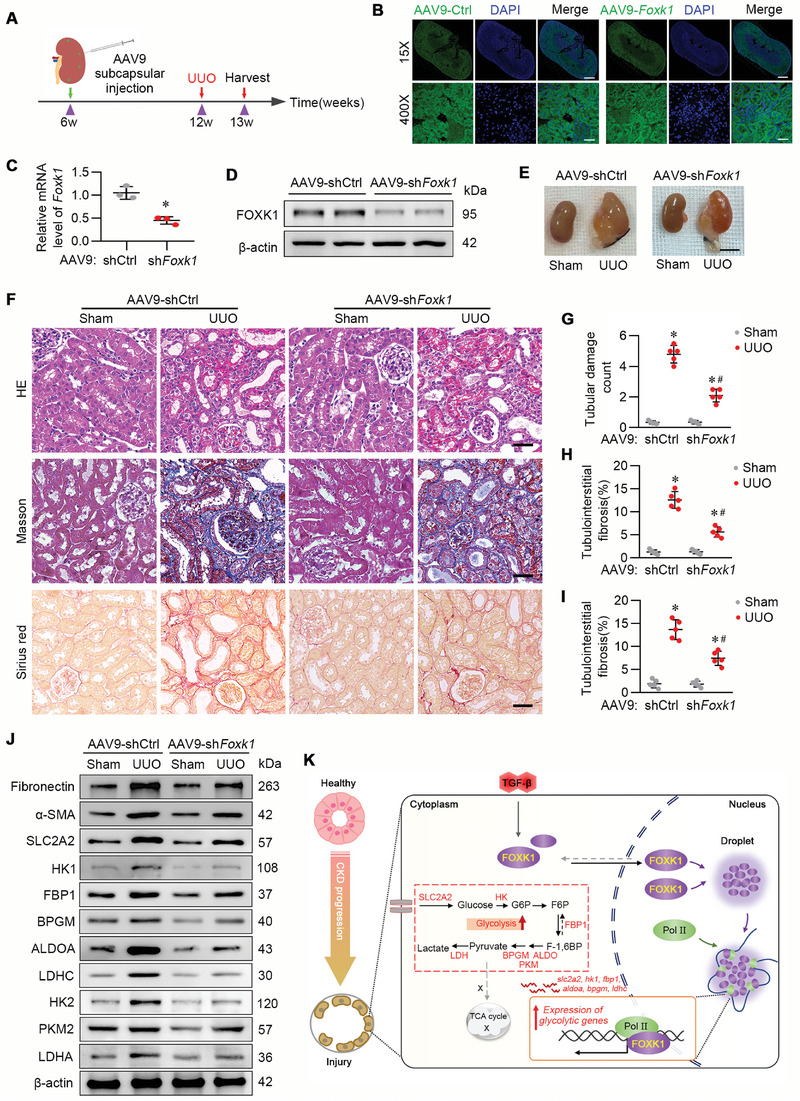
AAV9‐mediated knockdown of renal Foxk1 mitigated kidney fibrosis in UUO mice model. A) Schematic diagram of FOXK1 knockdown in mice. Renal subcapsular delivery of the AAV9‐shCtrl or AAV9‐sh*Foxk1* to wild‐type C57BL/6 mice at 6 weeks of age. After the delivery for six weeks, the mice were subjected to UUO surgery. B) Fluorescence microscopic analysis of EGFP in frozen sections of mouse kidney at 1 week after injection of AAV9‐shCtrl or AAV9‐ sh*Foxk1*. Nuclei were stained with DAPI (blue). Scale bar = 50 µm. C) qRT‐PCR analysis of *Foxk1* mRNA abundance in the whole kidney of AAV9‐shCtrl or AAV9‐shFoxk1 mice. D) WB analysis of FOXK1 in the whole kidney of AAV9‐shCtrl or AAV9‐ sh*Foxk1* mice. E) Gross appearance of kidneys from the indicated groups. Scale bar = 5 mm. F) H&E, Masson staining, and Sirius red staining were applied to examine the tubular damage and tubulointerstitial fibrosis percentage in the renal tissue section from the indicated groups. Scale Bar = 50 µm. *n* = 5 mice per group. G–I) Quantification of tubular damage score (G), and tubulointerstitial fibrosis percentage (H, I). *n* = 5 mice per group. Quantitative data are expressed as the mean ± S.E.M. *p < 0.05, compared to the sham group; *^#^p *< 0.05 compared with AAV9‐shCtrl‐UUO mice. J) Western blotting of the protein expression of the related molecules in kidney tissues from the indicated group. K) Schematic illustration of the role and mechanism of FOXK1‐triggered energic metabolism rewiring in TECs in the context of renal fibrosis. Transcriptional factor FOXK1 is specifically induced in proximal TECs as a response to insults such as insufficient oxygen supply, or fibrogenic factor TGF‐β1 stimulations. FOXK1 proteins translocated and enriched in the nucleus condensate into liquid droplets via the mechanism of LLPS. The RNA PoI II and the target gene motifs can be sequestrated together with FOXK1 in the droplets, resulting in the formation of membrane‐less compartments which allow FOXK1 to exercise the transcriptional activities with high efficacy. Through this mechanism, FOXK1 promotes the expression of a series of glycolysis‐related genes, such as *Slc2a2, Hk1, Fbp1, Aldoa, Bpgm, Ldhc*, and drives the shift of energy supply from FAO to glycolysis in TECs, which in turn aggravates renal fibrosis in CKD progression.

Our findings showing that TECs‐specific deletion of the *Foxk1* curbed the development of renal fibrosis enlighten the therapeutic potential of targeting FOXK1 in CKD treatment. We, therefore, explored a gene therapy strategy with adeno‐associated virus (AAV) serotype 9 (AAV9) as the gene delivery tool via renal SC administration, as described previously.^[^ ^42^, ^43^ ^]^ AAV9 carrying *Foxk1*‐specific short hairpin RNA under *Ksp‐cadherin* promoter direction (AAV9‐sh*Foxk1*) or AAV9‐shCtrl were constructed and administered by renal SC injection in 6‐week‐old mice. The mice were subjected to UUO or IRI operation at the indicated time (**Figure** **8**A; Figure S8A, Supporting Information). We collected the whole kidney to detect the efficiency of AAV9 delivery (Figure 8B). The mRNA and protein expression of FOXK1 were significantly decreased in the kidneys after injection of AAV9‐sh*Foxk1* for 6 weeks (Figure 8C,D). In the UUO and IRI murine models, a notable alleviation of renal fibrosis was observed in the AAV9‐sh*Foxk1*‐treated mice but not the AAV‐shCtrl mice (Figure 8E,F; Figure S8B,C, Supporting Information), manifested by the accumulation of extracellular matrix (Figure 8G–I; Figure S8D–F, Supporting Information) and fibrotic markers (Fibronectin and α‐SMA) expression (Figure 8J; Figure S8G, Supporting Information, and Figure S9, Supporting Information). In addition, glycolysis intensity and glycolysis‐related transcripts were significantly downregulated in AAV9‐sh*Foxk1* treated mice (Figure 8J; Figure S8G, Supporting Information, and Figure S9, Supporting Information). These results suggested that FOXK1‐targeted gene therapy is promising in CKD treatment by preventing glycolysis and fibrotic lesions in diseased kidneys.

## 3 Discussion

Renal fibrosis is a common pathological outcome of progressive kidney diseases. Although several pharmacological agents have emerged recently, the ideal regimen with promising therapeutic effects for CKD is still in dire need at present.^[^ ^44^ ^]^ Success in new therapy development mainly depends on an in‐depth understanding of the mechanism of renal fibrosis. In this study, we identified a novel fibrogenic factor, transcription factor FOXK1, which featured proximal tubule‐specific induction and a critical role in boosting glycolysis in TECs and promoting renal fibrosis. The major highlights of this study are as follows: 1) we depicted the expressional feature of FOXK1 in the context of kidney fibrosis progression; 2) we found that FOXK1 aggravates renal fibrosis partly due to its role in triggering glycolysis in proximal TECs; 3) we identified the glycolysis‐related genes regulated by FOXK1 and deciphered the mechanism of LLPS‐mediated transcription, including the corresponding IDR and the subunit of RNA Pol II involved (Figure 8K); 4) we also explored the potential of FOXK1‐targeted gene therapy in CKD treatment.

Cell metabolic alteration is an adaptive or maladaptive response to insults or harmful environmental changes. Renal tubular cells utilize different substrates, such as glucose, amino acids, and fatty acids, depending on the nephron segment, to generate energy through FAO and glycolysis.^[^ ^45^, ^46^ ^]^ Usually, proximal tubule cells adopt FAO as the preferred metabolic pathway to meet their energy demands. However, the metabolism switch from FAO to glycolysis is triggered in renal tubules under insufficient oxygen supply and toxic exposure.^[^ ^14^, ^47^ ^]^ Several studies have investigated the mechanism of the impaired capacity of FAO in renal tubules under pathological conditions of either AKI or CKD.^[^ ^47^ ^]^ However, how the pathological glycolytic shift is triggered in the tubule, and the key regulators responsible for this induction remain unclear, and it is imperative to comprehensively understand the energy transition in tubules since FAO and glycolysis are independent metabolism pathways. In this study, we identified FOXK1 as the master regulator of glycolysis in TECs under pathogenic conditions. FOXK1 is weakly expressed in kidney tissue under normal conditions but can be induced in renal tubules, predominantly the proximal TECs, in conditions of progressive CKD. An elevated FOXK1 expression is necessary to turn on glycolysis in TECs and may lead to the development of renal fibrosis. FOXK1 was initially discovered to regulate embryonic development and muscle formation^[^ ^48^ ^]^ and was later identified as a transcriptional factor regulating large arrays of target genes involved in multiple cellular processes, including metabolism, proliferation, cell signaling, apoptosis, and DNA damage.^[^ ^25^, ^32^ ^]^ Recent findings have documented its critical role in driving aerobic glycolysis by upregulating several enzymes involved in glycolysis.^[^ ^27^ ^]^ In addition, FOXK2, another Fox family member, was found to promote pulmonary fibrosis by enhancing glycolysis in fibroblasts.^[^ ^49^ ^]^ These findings show that FOXK1 may act as a fibrogenic factor in renal fibrosis by redirecting multiple cellular processes.

The cellular metabolism phenotype is closely associated with the cell fate and state. Renal tubules exhibit dynamic alteration in energy metabolic program throughout the disease course as either AKI or CKD and with great heterogeneity in terms of the nephron segment.^[^ ^28^, ^30^, ^50^ ^]^ The intricacies of energy reprogramming in tubules are derived from the difference in cell conditions undergoing injuries, which can be classified to the cell population of the healthy, acute injury, repairing, and failed repair. For proximal renal tubules, the metabolic transition from FAO to glycolysis is mainly present in the failed repair population, signaling the disease progress.^[^ ^51^, ^52^ ^]^ Our present study has identified FOXK1 as the key driver of glycolysis in TECs in the context of renal fibrosis, whether it is the same case for FOXK1 acts in AKI, another disease setting, needs further investigation. However, our studies revealed high consistency not only between the FOXK1 dynamic expression and the cell fate evolution (Figure 1A,B) but also between the FOXK1 cell‐type distribution and the glycolysis intensity level (Figure 1N), regardless of the AKI or UUO disease model. In addition, various insults such as the acute insufficiency of oxygen supply, inflammation, and TGF‐β1 activation have been found able to spur the glycolysis in TECs^[^ ^14^, ^18^, ^53^, ^54^, ^55^ ^]^ TGF‐β1 is the powerful inducer of FOXK1 expression and activation in different cells, including gastric cancer cells, hepatocyte.^[^ ^20^, ^56^, ^57^ ^]^ These findings lead us to speculate that the initiation of FOXK1 expression is the common pathway leading to the metabolic shift to glycolysis in TECs under distinct conditions.

The concomitant occurrence of an FAO decline and a glycolytic surge in TECs that signals an energy‐related metabolic reprogramming in the fibrotic kidney indicates that these two metabolic pathways function in a coordinated manner, and this may be ascribed to the presence of FAO and glycolysis co‐regulators. In this context, HNF4a or estrogen‐related receptor alpha (ESRRA) is modified during AKI.^[^ ^58^ ^]^ FOXK1 has multiple effects on cellular metabolism through transcriptional regulation of a panel of target genes.^[^ ^27^, ^31^, ^57^ ^]^ We herein report that FOXK1 boosts glycolysis in TECs by promoting a set of gene expressions, including *Slc2a2, Hk1, Fbp1, Aldoa, Bpgm, Ldhc, Hk2*, *Pkm2*, and *Ldha*. Previous studies from others have documented the capacity of FOXK1 to bolster FAO in preadipocytes via regulating relevant genes.^[^ ^27^, ^31^ ^]^ It raises the question of whether FOXK1 orchestrates two metabolism pathways of FAO and glycolysis in TECs concomitantly in the context of renal fibrosis, which needs to be addressed in the future.

LLPS, a fundamental biochemical process, is found present in cells through which intracellular molecules concentrate into droplets.^[^ ^36^ ^]^ Moreover, LLPS is essential for transcriptional factors to exercise transcriptional activities and thus regulates multiple cell functions.^[^ ^59^, ^60^, ^61^, ^62^ ^]^ Targeting the biomolecular condensation and protein aggregation to disrupt the LLPS may offer a promising approach for cancer therapy,^[^ ^63^ ^]^ inhibition of SARS‐CoV‐2 replication,^[^ ^64^ ^]^ and neurodegenerative disease.^[^ ^60^ ^]^ A recent report found that FOXK1 interacts with LSD1^[^ ^65^ ^]^ and increases its nuclear retention, while purified LSD1 protein forms phase‐separated liquid compartments directly in the phase‐separation buffer.^[^ ^65^, ^66^ ^]^ LLPS is primarily driven by IDRs, which are present in FOXK1 molecules with larger stretches. In this study, we verified that the glycolysis‐promoting role of FOXK1 depended on the LLPS capability. To the best of our knowledge, this is the first report on the nature of LLPS with FOXK1, as well as the structural basis of FOXK1 droplet formation. Moreover, we identified the 210–303aa region of FOXK1, the IDR responsible for liquid droplet formation and facilitating gene transcription. Recent observations suggest that RNA Pol II may be recruited to dynamic droplets via phase separation to interact with other transcription factors and coactivators to drive target genes' transcription.^[^ ^41^, ^67^, ^68^ ^]^ Similarly, we confirmed the formation of transcriptional condensates comprising RNA Pol II and FOXK1, and found that RNA Pol II orchestrates co‐transcriptional events with FOXK1. It has been reported that the C‐terminal domain (CTD) of the largest subunit of RNA Pol II is a regulatory hub for transcription and RNA processing,^[^ ^69^ ^]^ and CTD phosphorylation regulates the incorporation of Pol II into different condensates formed by mediator or splicing factors.^[^ ^68^, ^70^ ^]^ In this study, we unveil an unexpected interaction between FOXK1 and another subunit of RNA Pol II, namely POLR2K, that defines the molecular and structural basis for RNA Pol II‐FOXK1 droplet formation. Further research is needed to investigate the role of the phase‐separated environment created by FOXK1 in the regulation of mediator, splicing factors, and CTD phosphorylation.

In summary, we identified a novel fibrogenic factor in renal fibrosis progression, the transcriptional factor FOXK1, which emerged in a manner of proximal TEC‐specific distribution and cell‐state relevance of injured and failed repair. FOXK1 plays a pivotal role in bolstering the energic metabolism switch from FAO to glycolysis in TECs and aggravating the progression of renal fibrosis. A set of genes related to glycolysis were found to be transcriptionally upregulated by FOXK1, which formed condensates via LLPS within the nucleus and interacted with the target gene motifs. We also explored the strategy of curbing renal fibrosis by FOXK1‐targeted gene silencing therapy.

## 4 Experimental Section

### Antibodies and Reagents

Please refer to Table S4S (Supporting Information) for details of antibodies and related reagents used in this study.

### Mouse and Animal Models

Transgenic mice were generated and maintained. To establish the UUO‐induced murine model of renal fibrosis, mice underwent ligation of the left ureter with 4‐0 silk suture at two locations and cutting to prevent urinary tract infection. The UUO control mice underwent sham surgery of the right ureter. Mice were sacrificed and kidneys were collected on day 7 post‐surgery. A progressive renal fibrosis model induced by IRI was developed as described before20. In brief, the left renal artery was clamped with a microvascular clamp for 30 min at 37 °C by a heating device, followed by reperfusion. For control mice, a sham operation was performed on the right kidney instead of its removal. The mice were sacrificed and analyzed at the indicated time points post‐surgery. The study examined both male and female animals and similar findings for both sexes were reported. All procedures were formally reviewed and approved by the Animal Ethics Review Board of Wuhan University, China (IACUC Approval Number: WDRM20220204B).

### Human Subjects

Human renal biopsy samples were collected from study participants at the Renmin Hospital of Wuhan University (Wuhan, China). Normal tissue samples adjacent to the tumor were obtained from patients with renal cancers who underwent nephrectomy at Renmin Hospital of Wuhan University. All patients provided informed consent before inclusion in this study and patients’ clinical biochemical information was extracted from medical records. The study was conducted in accordance with the Declaration of Helsinki and approved by the Ethics Committees of Renmin Hospital of Wuhan University for Clinical Research (IACUC Approval Number: WDRY2023‐K096).

### Cell Culture

Two types of proximal tubular epithelial cell lines including mouse BUMPT cells and human HK‐2 cells were stored at the Nephrology and Urology Research Institute of Wuhan University. Cells were cultured in DMEM/F12 medium with 10% fetal bovine serum and 1% penicillin‐streptomycin in a 37 °C incubator with 5% CO_2_. Cells were seeded in six‐well culture plates for transfection and treatment with or without 10 ng mL^−1^ TGF‐β1 for 24 h.

### Histology and Immunohistochemical (IHC) Staining

Kidney samples were fixed with 4% paraformaldehyde overnight at room temperature and then embedded in paraffin. Tissue sections (4 µm) were stained with Hematoxylin‐Eosin, Masson, and Sirius red according to the manufacturer's protocols for histological microscopic examination. For IHC staining, samples were incubated with horseradish peroxide (HRP) labeled secondary antibodies and DAB color development reagent. Slides were observed under a microscope (Olympus, Tokyo, Japan). Tubular damage count and the percentage of tubulointerstitial fibrosis were assessed as previously described.^[^ ^19^, ^20^ ^]^

### Cell Transfection

Cell transfection was performed according to the protocol of the manufacturer. Briefly, cells were incubated with *FOXK1* shRNA lentivirus (*FOXK1* shRNA sequence: 5′‐CUCUCUUUGAACCGUUACUTT‐3′. From Genechem Co., China.) for 16h. Transduced cells that expressed puromycin‐resistant genes were selected by puromycin (at 5 ng mL^−1^ for 2 days) on day 2. The expression of *FOXK1* was examined by qRT‐PCR and western blot.

### Immunofluorescence (IF) Staining

IF staining was conducted as described previously.^[^ ^19^, ^20^ ^]^ Paraffin‐embedded kidney tissue sections were deparaffinized, hydrated, antigen repaired, blocked, and then incubated with the indicated primary antibodies. For IF staining of cultured cells, coverslips‐bearing cells were fixed in 4% paraformaldehyde and blocked in phosphate‐buffered saline (PBS) containing 10% normal donkey serum, followed by incubation with the indicated primary antibodies. The samples were stained with secondary antibodies conjugated with Alexa Fluor 488 or 594 conjugation and DAPI. Images were acquired using a confocal laser microscope (FV1000; Olympus). The fluorescence intensity of IF staining was quantified by Image J analysis software.

### Western Blotting (WB)

WB was performed on whole‐cell or tissue lysates as described before.^[^ ^19^, ^20^ ^]^ Samples were extracted from the kidney cortex or cultured cells with protein lysis buffer containing a proteinase inhibitor cocktail and phosphatase inhibitor. Samples were loaded on 10% or 12% gels for sodium dodecyl sulfate‐polyacrylamide gel electrophoresis (SDS‐PAGE) and then transferred onto polyvinylidene fluoride (PVDF) membranes (Merck Millipore). PVDF membranes were blocked with 5% nonfat milk and incubated with indicated primary antibodies overnight at 4 °C, followed by incubation with HRP secondary antibodies at room temperature for 1 h. Blots were developed with chemiluminescence using ECL luminous fluid (Biology, Wuhan, China) and ChemiDoc XR Plus (Bio‐Rad Laboratories, Hercules, CA). One analysis software (Bio‐Rad Laboratories) was used to quantify the band intensity of indicated proteins.

### Immunoprecipitation (IP) Assays

The IP assay was performed according to the manufacturer's protocol (Merck Millipore). In brief, HK‐2 cells were lysed, and the cell lysates were precleared using protein A/G magnetic beads (Merck Millipore). Precleared lysates were immunoprecipitated by overnight incubation with anti‐FOXK1 antibodies at 4  °C with gentle rocking. The beads were washed, and the immune complexes were suspended in a loading buffer and then denatured at 100 °C. The immunoprecipitated proteins were resolved by SDS‐PAGE and analyzed by Western blotting as described above. An isotype‐specific IgG was applied as a negative control.

### Quantitative Reverse Transcriptase‐Polymerase Chain Reaction (qRT‐PCR)

Total RNA was extracted from kidney cortex samples or cells flowing the protocol of RNAiso plus kit (Takara, Japan). Total mRNA was reverse transcribed into cDNA using a Strand cDNA Synthesis Kit (Vazyme, China). The expression of target mRNA was quantified using SYBR qPCR Master Mix (Vazyme, China) with a CFX RT‐PCR Detection System (Bio‐Rad). To perform semi‐quantitative studies, β‐actin was used as an internal reference. The primer sequences are listed in Table S5, Supporting Information.

### Measurements of Lactate

The lactate levels in the cell culture medium were measured using the Lactate Assay Kit (Cayman, America) according to the manufacturer's protocol. And the lactate levels in the renal cortex were detected using the Lactate Assay Kit (Jiancheng Bioengineering Institute, Nanjing China).

### Bioenergetic Analysis

ECAR and OCR were measured by the XF24 Analyzer (Seahorse Bioscience) according to the instructions of the manufacturer (Seahorse Bioscience, Billerica, MA). The data of ECAR and OCR data were analyzed and plotted by using the XF24 software version 1.8 (Seahorse Bioscience). Data were normalized for total protein content per well.

### Chromatin Immunoprecipitation (ChIP) Assays and ChIP‐seq

ChIP assays were performed with a Pierce Magnetic ChIP kit (Thermo Scientific, Cat.# 26 157) according to the manufacturer's protocol. Briefly, cultured HK‐2 cells underwent indicated treatment and were fixed with 1% paraformaldehyde. After the lysis and MNase digestion, chromatin was sonicated on ice with three 20‐s pulses at 3 watts power (using Sonicator 3000 Instrument). Immunoprecipitation was performed overnight at 4 °C. The rabbit anti‐FOXK1 antibody (ab18196), anti‐IgG antibody, anti‐RNA Pol II antibody were used for ChIP. Protein A/G Magnetic Beads were added into each IP sample for 4 h incubation at 4 °C. After IP elution, DNA recovery, ChIP‐PCR, or ChIP seq were performed.

ChIP‐seq libraries were prepared and sequenced by Wuhan IGENEBOOK Biotechnology Co. Ltd.^[^ ^71^ ^]^ Raw Reads were transformed using the Illumina Platform. Trimmomatic (version: 0.36) software was applied to improve the quality of the data and get clean reads. The Clean reads were aligned to the human genome (hg19) using BWA software (version: 0.7.15‐r1140). To identify a significant binding site of FOXK1, peak calling was performed with MACS (version: 2.1.1), with the input used as the control. Peak annotation was performed with BEDTools (Version v2.25.0). Motif analysis was performed with Homer (version 3). Primers for ChIP‐PCR are listed in Table S5, Supporting Information.

### Online Public Database Analysis

Two publicly available resources were used for transcriptomic analysis. 1) Kidney interactive transcriptomics platform (http://humphreyslab.com/SingleCell/), which was developed by Dr. Humphreys. The results generated in this study used data sources (GSE131882, GSE151302, and GSE190887) that have been published previously.^[^ ^28^, ^29^, ^30^ ^]^ 2) Nephroseq (www.nephroseq.org), a platform of comprehensive renal disease gene expression data sets.^[^ ^72^ ^]^ The expression of FOXK1 in kidney tissues of patients with CKD was predicted based on the Nephroseq database (https://www.nephroseq.org, University of Michigan, O'Brien Renal Center) which contains gene expression data of patients with CKD. Minimum fold change was set at 1.5 and probability p values were considered significant when *p * <  0.05.

Data presented in this study received written permission from both resources mentioned earlier. Specifically, Figure 1B and Figure 1D were created using the kidney interactive transcriptomics platform. Figure 1C was generated using the Nephroseq database.

### Droplet Assays

Recombinant FOXK1‐mEGFP or mEGFP fusion proteins were diluted to the indicated concentrations and 10% PEG‐8000 (polyethylene glycol, molecular weight 8000) was added. Then the solution was dropped onto a glass slide with a cover glass. Imaging was performed with a fluorescence microscope (Olympus).

### Statistics

Data were presented as mean ± S.E.M from at least three biological replicates of experiments. Statistical significance differences were calculated by Student's t‐test and one‐way ANOVA Tukey's post‐test. *p *< 0.05 was considered statistically significant. All statistical analysis was performed by the GraphPad Prism software and the Statistical Program for Social Sciences software.

### Ethical Statement

All protocols were approved by the Animal Ethics Review Board of Wuhan University (IACUC Issue NO. WDRM20220204B) and the Ethics Committees of Renmin Hospital of Wuhan University for Clinical Research (IACUC Issue NO. WDRY2023‐K096). And all protocols are performed in accordance with the guidelines of the National Health and Medical Research Council of China.

## Conflict of Interest

The authors declare no conflict of interest.

## Author Contributions

L.Z. and M.T. contributed equally to this work. L.Z., M.T., and H.W. conceived the project and designed the experiments. H.G., L.L., and G.D. advised on the design of experiments. M.T., L.Z., M.Z., X.W., Y.L., and C.L. performed the main experiments and analyzed data. Y.W., M.T., and L.Z. collected and analyzed the human kidney samples. J.H., C.C., X.C., and W.L. reviewed and analyzed the data. L.Z. and M.T. drafted the manuscript. L.Z. and H.W. revised the manuscript. All authors read and approved the final manuscript.

## Supporting information

Supporting Information

## Data Availability

The data that support the findings of this study are available from the corresponding author upon reasonable request.

## References

[1] J. S. Duffield , J. Clin. Invest. 2014, 124, 2299. 24892703 10.1172/JCI72267 PMC4038570

[2] A. A. Eddy , Kidney Int. Suppl. 2014, 4, 2. 10.1038/kisup.2014.2 PMC4220516 25401038

[3] B. D. Humphreys , Ann. Rev. Physiol. 2018, 80, 309. 29068765 10.1146/annurev-physiol-022516-034227

[4] Y. Liu , Nat. Rev. Nephrol. 2011, 7, 684. 22009250 10.1038/nrneph.2011.149 PMC4520424

[5] L. S. Gewin , Matrix Biol. 2018, 68, 248. 29425694 10.1016/j.matbio.2018.02.006 PMC6015527

[6] N. Yamashita , R. Kramann , TEM 2024, 35, 31. 37775469 10.1016/j.tem.2023.09.001

[7] V. Miguel , R. Kramann , FEBS Open Bio. 2023, 13, 1154. 10.1002/2211-5463.13568 PMC10315765 36723270

[8] H. Scholz , F. J. Boivin , K. M. Schmidt‐Ott , S. Bachmann , K. U. Eckardt , U. I. Scholl , P. B. Persson , Nat. Rev. Nephrol. 2021, 17, 335. 33547418 10.1038/s41581-021-00394-7

[9] B. B. Lake , R. Menon , S. Winfree , Q. Hu , R. Melo Ferreira , K. Kalhor , D. Barwinska , E. A. Otto , M. Ferkowicz , D. Diep , N. Plongthongkum , A. Knoten , S. Urata , L. H. Mariani , A. S. Naik , S. Eddy , B. Zhang , Y. Wu , D. Salamon , J. C. Williams , X. Wang , K. S. Balderrama , P. J. Hoover , E. Murray , J. L. Marshall , T. Noel , A. Vijayan , A. Hartman , F. Chen , S. S. Waikar , et al., Nature 2023, 619, 585. 37468583 10.1038/s41586-023-05769-3 PMC10356613

[10] L. Wen , Y. Li , S. Li , X. Hu , Q. Wei , Z. Dong , Front. Med. 2021, 8, 744122. 10.3389/fmed.2021.744122 PMC8666949 34912819

[11] H. M. Kang , S. H. Ahn , P. Choi , Y. A. Ko , S. H. Han , F. Chinga , A. S. Park , J. Tao , K. Sharma , J. Pullman , E. P. Bottinger , I. J. Goldberg , K. Susztak , Nat. Med. 2015, 21, 37. 25419705 10.1038/nm.3762 PMC4444078

[12] R. Frazier , R. Mehta , X. Cai , J. Lee , S. Napoli , T. Craven , J. Tuazon , A. Safdi , J. Scialla , K. Susztak , T. Isakova , Kidney Int. Rep. 2019, 4, 94. 30596172 10.1016/j.ekir.2018.09.006 PMC6308372

[13] A. Poyan Mehr , M. T. Tran , K. M. Ralto , D. E. Leaf , V. Washco , J. Messmer , A. Lerner , A. Kher , S. H. Kim , C. C. Khoury , S. J. Herzig , M. E. Trovato , N. Simon‐Tillaux , M. R. Lynch , R. I. Thadhani , C. B. Clish , K. R. Khabbaz , E. P. Rhee , S. S. Waikar , A. H. Berg , S. M. Parikh , Nat. Med. 2018, 24, 1351. 30127395 10.1038/s41591-018-0138-z PMC6129212

[14] R. Lan , H. Geng , P. K. Singha , P. Saikumar , E. P. Bottinger , J. M. Weinberg , M. A. Venkatachalam , JASN 2016, 27, 3356. 27000065 10.1681/ASN.2015020177 PMC5084876

[15] A. Bataille , P. Galichon , N. Chelghoum , B. M. Oumoussa , M. J. Ziliotis , I. Sadia , S. Vandermeersch , N. Simon‐Tillaux , D. Legouis , R. Cohen , Y. C. Xu‐Dubois , M. Commereuc , E. Rondeau , S. Le Crom , A. Hertig , Cell. Physiol.Biochem. 2018, 47, 1338. 29929186 10.1159/000490819

[16] R. Lakhia , M. Yheskel , A. Flaten , E. B. Quittner‐Strom , W. L. Holland , V. Patel , Am. J. Physiol. Renal Physiol. 2018, 314, F122. 28903946 10.1152/ajprenal.00352.2017 PMC5866355

[17] L. Chen , R. P. Vasoya , N. H. Toke , A. Parthasarathy , S. Luo , E. Chiles , J. Flores , N. Gao , E. M. Bonder , X. Su , M. P. Verzi , Gastroenterology 2020, 158, 985. 31759926 10.1053/j.gastro.2019.11.031 PMC7062567

[18] D. R. Lemos , M. McMurdo , G. Karaca , J. Wilflingseder , I. A. Leaf , N. Gupta , T. Miyoshi , K. Susa , B. G. Johnson , K. Soliman , G. Wang , R. Morizane , J. V. Bonventre , J. S. Duffield , JASN 2018, 29, 1690. 29739813 10.1681/ASN.2017121283 PMC6054344

[19] Q. Yan , K. Zhu , L. Zhang , Q. Fu , Z. Chen , S. Liu , D. Fu , R. Nakazato , K. Yoshioka , B. Diao , G. Ding , X. Li , H. Wang , Commun. Biol. 2020, 3, 288. 32504044 10.1038/s42003-020-1008-z PMC7275040

[20] M. Tian , L. Zhang , M. Zhang , L. Qiao , B. Xu , C. Li , S. Liu , Y. Song , Z. Wei , Y. Wang , H. Wang , iScience 2023, 26, 106396. 37013185 10.1016/j.isci.2023.106396 PMC10066564

[21] P. Rao , M. Pang , X. Qiao , H. Yu , H. Wang , Y. Yang , X. Ren , M. Hu , T. Chen , Q. Cao , Y. Wang , M. Khushi , G. Zhang , Y. M. Wang , C. Heok P'ng , B. Nankivell , V. W. Lee , S. I. Alexander , G. Zheng , D. C. Harris , Lab. Investigat. J. Tech. Methods Pathol. 2019, 99, 1689. 10.1038/s41374-019-0276-z 31243340

[22] Z. Xin , Z. Ma , W. Hu , S. Jiang , Z. Yang , T. Li , F. Chen , G. Jia , Y. Yang , Ageing Res. Rev. 2018, 41, 42. 29138094 10.1016/j.arr.2017.11.002

[23] M. Black , D. Milewski , T. Le , X. Ren , Y. Xu , V. V. Kalinichenko , T. V. Kalin , Cell Rep. 2018, 23, 442. 29642003 10.1016/j.celrep.2018.03.067 PMC5947867

[24] N. Miyashita , M. Horie , H. I. Suzuki , M. Saito , Y. Mikami , K. Okuda , R. C. Boucher , M. Suzukawa , A. Hebisawa , A. Saito , T. Nagase , Am. J. Resp. Cell Mol. Biol. 2020, 63, 831. 10.1165/rcmb.2019-0396OC PMC8017595 32946266

[25] Y. Feng , Z. Bai , J. Song , Z. Zhang , Mol. Med. Rep. 2021, 23. 10.3892/mmr.2020.11730 PMC7723167 33300075

[26] C. J. Bowman , D. E. Ayer , B. D. Dynlacht , Nat. Cell Biol. 2014, 16, 1202. 25402684 10.1038/ncb3062 PMC4250422

[27] V. Sukonina , H. Ma , W. Zhang , S. Bartesaghi , S. Subhash , M. Heglind , H. Foyn , M. J. Betz , D. Nilsson , M. E. Lidell , J. Naumann , S. Haufs‐Brusberg , H. Palmgren , T. Mondal , M. Beg , M. P. Jedrychowski , K. Taskén , A. Pfeifer , X. R. Peng , C. Kanduri , S. Enerbäck , Nature 2019, 566, 279. 30700909 10.1038/s41586-019-0900-5

[28] H. Li , E. E. Dixon , H. Wu , B. D. Humphreys , Cell Metab. 2022, 34, 1977. 36265491 10.1016/j.cmet.2022.09.026 PMC9742301

[29] P. C. Wilson , H. Wu , Y. Kirita , K. Uchimura , N. Ledru , H. G. Rennke , P. A. Welling , S. S. Waikar , B. D. Humphreys , Proc. Nat. Acad. Sci. 2019, 116, 19619. 31506348 10.1073/pnas.1908706116 PMC6765272

[30] Y. Muto , P. C. Wilson , N. Ledru , H. Wu , H. Dimke , S. S. Waikar , B. D. Humphreys , Nat. Commun. 2021, 12, 2190. 33850129 10.1038/s41467-021-22368-w PMC8044133

[31] M. Sakaguchi , W. Cai , C. H. Wang , C. T. Cederquist , M. Damasio , E. P. Homan , T. Batista , A. K. Ramirez , M. K. Gupta , M. Steger , N. J. Wewer Albrechtsen , S. K. Singh , E. Araki , M. Mann , S. Enerbäck , C. R. Kahn , Nat. Commun. 2019, 10, 1582. 30952843 10.1038/s41467-019-09418-0 PMC6450906

[32] Y. Liu , W. Ding , H. Ge , M. Ponnusamy , Q. Wang , X. Hao , W. Wu , Y. Zhang , W. Yu , X. Ao , J. Wang , Cancer Lett. 2019, 458, 1. 31132431 10.1016/j.canlet.2019.05.030

[33] H. Cao , J. Luo , Y. Zhang , X. Mao , P. Wen , H. Ding , J. Xu , Q. Sun , W. He , C. Dai , K. Zen , Y. Zhou , J. Yang , L. Jiang , Kidney Int. 2020, 98, 686. 32739207 10.1016/j.kint.2020.03.035

[34] H. Ding , L. Jiang , J. Xu , F. Bai , Y. Zhou , Q. Yuan , J. Luo , K. Zen , J. Yang , Am. J. Physiol. Renal Physiol. 2017, 313, F561. 28228400 10.1152/ajprenal.00036.2017

[35] J. Li , H. Liu , S. Takagi , K. Nitta , M. Kitada , S. P. Srivastava , Y. Takagaki , K. Kanasaki , D. Koya , JCI Insight 2020, 5. 10.1172/jci.insight.129034 PMC7213787 32134397

[36] S. Alberti , A. Gladfelter , T. Mittag , Cell 2019, 176, 419. 30682370 10.1016/j.cell.2018.12.035 PMC6445271

[37] H. Zhang , X. Ji , P. Li , C. Liu , J. Lou , Z. Wang , W. Wen , Y. Xiao , M. Zhang , X. Zhu , Sci. China. Life Sci. 2020, 63, 953. 32548680 10.1007/s11427-020-1702-x

[38] G. G. Fuller , J. K. Kim , J. Cell Sci. 2021, 134. 10.1242/jcs.258469 PMC8572002 34668544

[39] Y. G. Zhao , H. Zhang , Dev. Cell 2020, 55, 30. 32726575 10.1016/j.devcel.2020.06.033

[40] H. Lu , D. Yu , A. S. Hansen , S. Ganguly , R. Liu , A. Heckert , X. Darzacq , Q. Zhou , Nature 2018, 558, 318. 29849146 10.1038/s41586-018-0174-3 PMC6475116

[41] Y. Lu , T. Wu , O. Gutman , H. Lu , Q. Zhou , Y. I. Henis , K. Luo , Nat. Cell Biol. 2020, 22, 453. 32203417 10.1038/s41556-020-0485-0 PMC11044910

[42] S. Chen , H. Huang , Y. Liu , C. Wang , X. Chen , Y. Chang , Y. Li , Z. Guo , Z. Han , Z. C. Han , Q. Zhao , X. M. Chen , Z. Li , iScience 2021, 24, 103243. 34746706 10.1016/j.isci.2021.103243 PMC8554536

[43] Q. Q. Chen , K. Liu , N. Shi , G. Ma , P. Wang , H. M. Xie , S. J. Jin , T. T. Wei , X. Y. Yu , Y. Wang , J. Y. Zhang , P. Li , L. W. Qi , L. Zhang , Nat. Commun. 2023, 14, 1713. 36973294 10.1038/s41467-023-37450-8 PMC10043283

[44] R. Huang , P. Fu , L. Ma , Signal Tran. Target.Ther. 2023, 8, 129. 10.1038/s41392-023-01379-7 PMC10023808 36932062

[45] T. J. Rabelink , M. Giera , Nat. Rev. Nephrol. 2019, 15, 596. 31388129 10.1038/s41581-019-0192-x

[46] S. Uchida , H. Endou , Am. J. Physiol. 1988, 255, F977. 2847554 10.1152/ajprenal.1988.255.5.F977

[47] A. Faivre , T. Verissimo , H. Auwerx , D. Legouis , S. de Seigneux , Front. Med. 2021, 8, 742072. 10.3389/fmed.2021.742072 PMC8585753 34778303

[48] D. J. Garry , Q. Yang , R. Bassel‐Duby , R. S. Williams , Devel. Biol. 1997, 188, 280. 9268575 10.1006/dbio.1997.8657

[49] Q. Xu , D. Cheng , G. Li , Y. Liu , P. Li , W. Sun , D. Ma , C. Ni , Int. J. Biol. Sci. 2021, 17, 2294. 34239356 10.7150/ijbs.57915 PMC8241722

[50] Y. Kirita , H. Wu , K. Uchimura , P. C. Wilson , B. D. Humphreys , Proc. Natl. Acad. Sci. U.S.A. 2020, 117, 15874. 32571916 10.1073/pnas.2005477117 PMC7355049

[51] J. D. Rabinowitz , G. M. Mutlu , Natu. Metabol. 2019, 1, 12.

[52] G. Garibotto , A. Sofia , S. Saffioti , A. Bonanni , I. Mannucci , D. Verzola , Clin. Nutr. 2010, 29, 424. 20207454 10.1016/j.clnu.2010.02.005

[53] G. Z. Quinn , P. Dhillon , K. Susztak , Semin. Nephrol. 2020, 40, 199. 32303282 10.1016/j.semnephrol.2020.01.010 PMC7682750

[54] W. Hua , P. Ten Dijke , S. Kostidis , M. Giera , M. Hornsveld , CMLS 2020, 77, 2103. 31822964 10.1007/s00018-019-03398-6 PMC7256023

[55] J. Soukupova , A. Malfettone , P. Hyroššová , M. I. Hernández‐Alvarez , I. Peñuelas‐Haro , E. Bertran , A. Junza , J. Capellades , G. Giannelli , O. Yanes , A. Zorzano , J. C. Perales , I. Fabregat , Sci. Rep. 2017, 7, 12486. 28970582 10.1038/s41598-017-12837-y PMC5624948

[56] Y. Peng , P. Zhang , X. Huang , Q. Yan , M. Wu , R. Xie , Y. Wu , M. Zhang , Q. Nan , J. Zhao , A. Li , J. Xiong , Y. Ren , Y. Bai , Y. Chen , S. Liu , J. Wang , Cell Death Dis. 2016, 7, e2480. 27882939 10.1038/cddis.2016.225 PMC5260906

[57] S. Fujinuma , H. Nakatsumi , H. Shimizu , S. Sugiyama , A. Harada , T. Goya , M. Tanaka , M. Kohjima , M. Takahashi , Y. Izumi , M. Yagi , D. Kang , M. Kaneko , M. Shigeta , T. Bamba , Y. Ohkawa , K. I. Nakayama , Cell Rep. 2023, 42, 112530. 37209098 10.1016/j.celrep.2023.112530

[58] D. Legouis , S. E. Ricksten , A. Faivre , T. Verissimo , K. Gariani , C. Verney , P. Galichon , L. Berchtold , E. Feraille , M. Fernandez , S. Placier , K. Koppitch , A. Hertig , P. Y. Martin , M. Naesens , J. Pugin , A. P. McMahon , P. E. Cippà , S. de Seigneux , Nat. Metabol. 2020, 2, 732. 10.1038/s42255-020-0238-1 32694833

[59] B. Wang , L. Zhang , T. Dai , Z. Qin , H. Lu , L. Zhang , F. Zhou , Signal Transduct. Target. Ther. 2021, 6, 290. 34334791 10.1038/s41392-021-00678-1 PMC8326283

[60] S. Boyko , W. K. Surewicz , Trends Cell Biol. 2022, 32, 611. 35181198 10.1016/j.tcb.2022.01.011 PMC9189016

[61] Z. Zong , F. Xie , S. Wang , X. Wu , Z. Zhang , B. Yang , F. Zhou , Cell 2024, 187, 2375. 38653238 10.1016/j.cell.2024.04.002

[62] Y. Jiang , J. Gu , X. Niu , J. Hu , Y. Zhang , D. Li , Y. Tang , C. Liu , Z. Li , Circulation 2024. 10.1161/CIRCULATIONAHA.123.067519 PMC11404759 38328928

[63] J. L. Silva , D. Foguel , V. F. Ferreira , T. Vieira , M. A. Marques , G. D. S. Ferretti , T. F. Outeiro , Y. Cordeiro , G. A. P. de Oliveira , Chem. Rev. 2023, 123, 9094. 37379327 10.1021/acs.chemrev.3c00131

[64] S. Wang , T. Dai , Z. Qin , T. Pan , F. Chu , L. Lou , L. Zhang , B. Yang , H. Huang , H. Lu , F. Zhou , Nat. Cell Biol. 2021, 23, 718. 34239064 10.1038/s41556-021-00710-0

[65] H. Araki , S. Hino , K. Anan , K. Kuribayashi , K. Etoh , D. Seko , R. Takase , K. Kohrogi , Y. Hino , Y. Ono , E. Araki , M. Nakao , eLife 2023, 12. 10.7554/eLife.84618 PMC9876571 36695573

[66] M. Xu , D. Senanayaka , R. Zhao , T. Chigumira , A. Tripathi , J. Tones , R. M. Lackner , A. R. Wondisford , L. N. Moneysmith , A. Hirschi , S. Craig , S. Alishiri , R. J. O'Sullivan , D. M. Chenoweth , N. J. Reiter , H. Zhang , Nat. Commun. 2024, 15, 2165. 38461301 10.1038/s41467-024-46509-z PMC10925046

[67] C. Guo , Z. Che , J. Yue , P. Xie , S. Hao , W. Xie , Z. Luo , C. Lin , Sci. Adv. 2020, 6, eaay4858. 32270036 10.1126/sciadv.aay4858 PMC7112754

[68] Y. E. Guo , J. C. Manteiga , J. E. Henninger , B. R. Sabari , A. Dall'Agnese , N. M. Hannett , J. H. Spille , L. K. Afeyan , A. V. Zamudio , K. Shrinivas , B. J. Abraham , A. Boija , T. M. Decker , J. K. Rimel , C. B. Fant , T. I. Lee , I. I. Cisse , P. A. Sharp , D. J. Taatjes , R. A. Young , Nature 2019, 572, 543. 31391587 10.1038/s41586-019-1464-0 PMC6706314

[69] L. M. Appel , V. Franke , M. Bruno , I. Grishkovskaya , A. Kasiliauskaite , T. Kaufmann , U. E. Schoeberl , M. G. Puchinger , S. Kostrhon , C. Ebenwaldner , M. Sebesta , E. Beltzung , K. Mechtler , G. Lin , A. Vlasova , M. Leeb , R. Pavri , A. Stark , A. Akalin , R. Stefl , C. Bernecky , K. Djinovic‐Carugo , D. Slade , Nat. Commun. 2021, 12, 6078. 34667177 10.1038/s41467-021-26360-2 PMC8526623

[70] S. Malik , R. G. Roeder , Nat. Rev. Genet. 2023, 24, 767. 37532915 10.1038/s41576-023-00630-9 PMC11088444

[71] Q. L. Wan , X. Meng , W. Dai , Z. Luo , C. Wang , X. Fu , J. Yang , Q. Ye , Q. Zhou , Sci. Adv. 2021, 7. 10.1126/sciadv.abc3026 PMC7775758 33523838

[72] S. Martini , F. Eichinger , V. Nair , M. Kretzler , Rev. End. Metabol. Dis. 2008, 9, 267. 10.1007/s11154-008-9103-3 PMC2597685 18704688

