# Supplementary material for: Forkhead Box Protein K1 Promotes Chronic Kidney Disease by Driving Glycolysis in Tubular Epithelial Cells
Source: Adv Sci (Weinh). 2024 Jul 31;11(36):2405325. doi: 10.1002/advs.202405325 (PMC11423168; doi:10.1002/advs.202405325)
Supplement: Supplementary file 1 — Supporting Information [file ADVS-11-2405325-s001.docx]

Supporting Information

Forkhead Box Protein K1 Promotes Chronic Kidney Disease by Driving Glycolysis in Tubular Epithelial Cells

*Lu Zhang, Maoqing Tian, Meng Zhang, Chen Li, Xiaofei Wang, Yuyu Long, Yujuan Wang, Jijia Hu, Cheng Chen, Xinghua Chen, Wei Liang, Guohua Ding, Hua Gan, Lunzhi Liu, Huiming Wang**

**List of Supporting Information**

**Supplemental Figures and Legends.**

1. **Figure S1. Schematic diagram of the generation of TEC-specific FOXK1 knockout mouse.**
2. **Figure S2. Tubular-specific Foxk1-deficient mice ameliorate kidney fibrosis in IRI model.**
3. **Figure S3. ChIP-PCR detection of FOXK1.** Related to Figure 4.
4. **Figure S4. FOXK1 enhances glycolysis related proteins expression in vitro.** Related to Figure 5.
5. **Figure S5. The levels of glycolysis related genes and lactate in fibrotic mice kidney.** Related to Figure 5.
6. **Figure S6. FOXK1 enhances glycolysis related proteins expression in fibrotic mice kidney.** Related to Figure 5.
7. **Figure S7. FOXK1-mEGFP increased the mRNA levels of FOXK1 targeted glycolytic genes.** Related to Figure 7.
8. **Figure S8. AAV9-mediated knockdown of renal FOXK1 ameliorate kidney fibrosis in IRI mice model.** Related to Figure 8.
9. **Figure S9. AAV9-mediated knockdown of renal FOXK1 downregulated kidney fibrotic markers in UUO mice model.** Related to Figure 8.

**Supplemental Tables.**

**1) Table S1. Baseline characteristics of CKD and adjacent normal tissues patients.**

**2) Table S2. Clinical information of renal obstruction patients.**

**3)** **Table S3. Genes are enriched in the glycolysis/gluconeogenesis pathway**

**4) Table S4. Resources list.**

**5) Table S5. Primer sequences used in this study.**


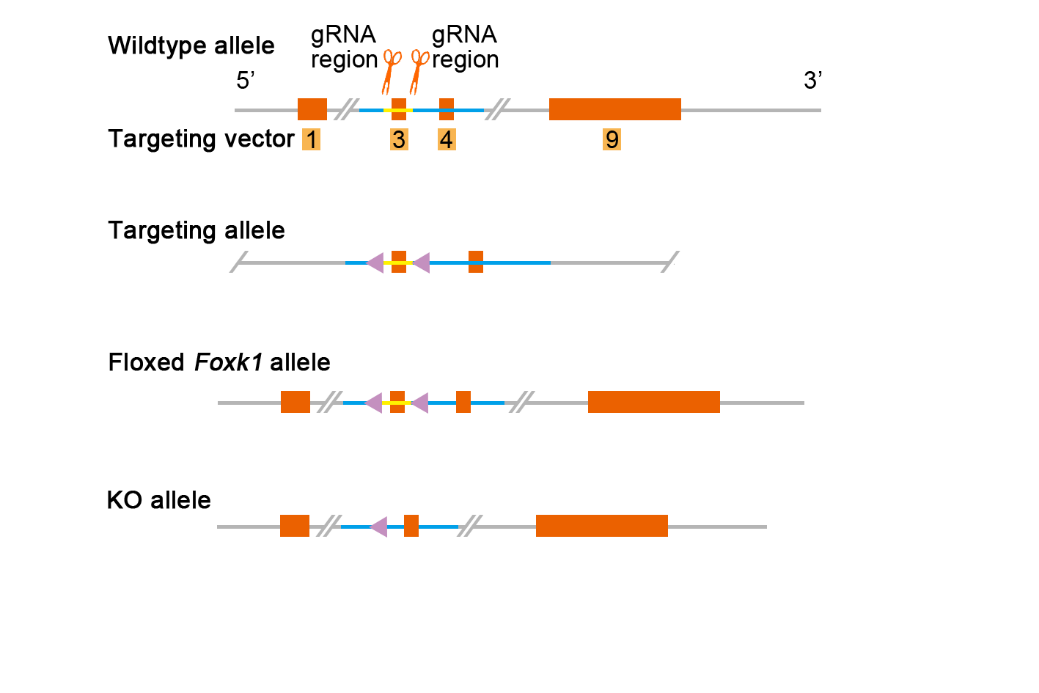
**Supplemental Figures.**

**Figure S1. Schematic diagram of the generation of TEC-specific *Foxk1* knockout mouse.** To generate inducible TECs-specific *Foxk1* deficient mice (*Foxk1*^cKO^), *Foxk1^flox/flox^* mice were cross-bred with *Ggt1-Cre* mice. Mice carrying mutant *Foxk1* and *Ggt1-Cre* alleles reproduced from heterozygous parents (*Foxk1^flox^*^/+^: *Cre+*). Efficacy of knockdown were tested by PCR genotyping, western blotting, and qRT-PCR. The *Foxk1-flox* and *Ggt1-Cre* alleles detected by PCR using the primers 5’-TACTAGAACCTCTCCTATTCACTTA-3’, 5’-TCCACAAGAGGGAATTTGAAAC-3’ and 5’-CATCACATCAGGCACCCCAGAA-3’, 5’-GAACATCTTCAGGTTCTGCGGGA-3’.


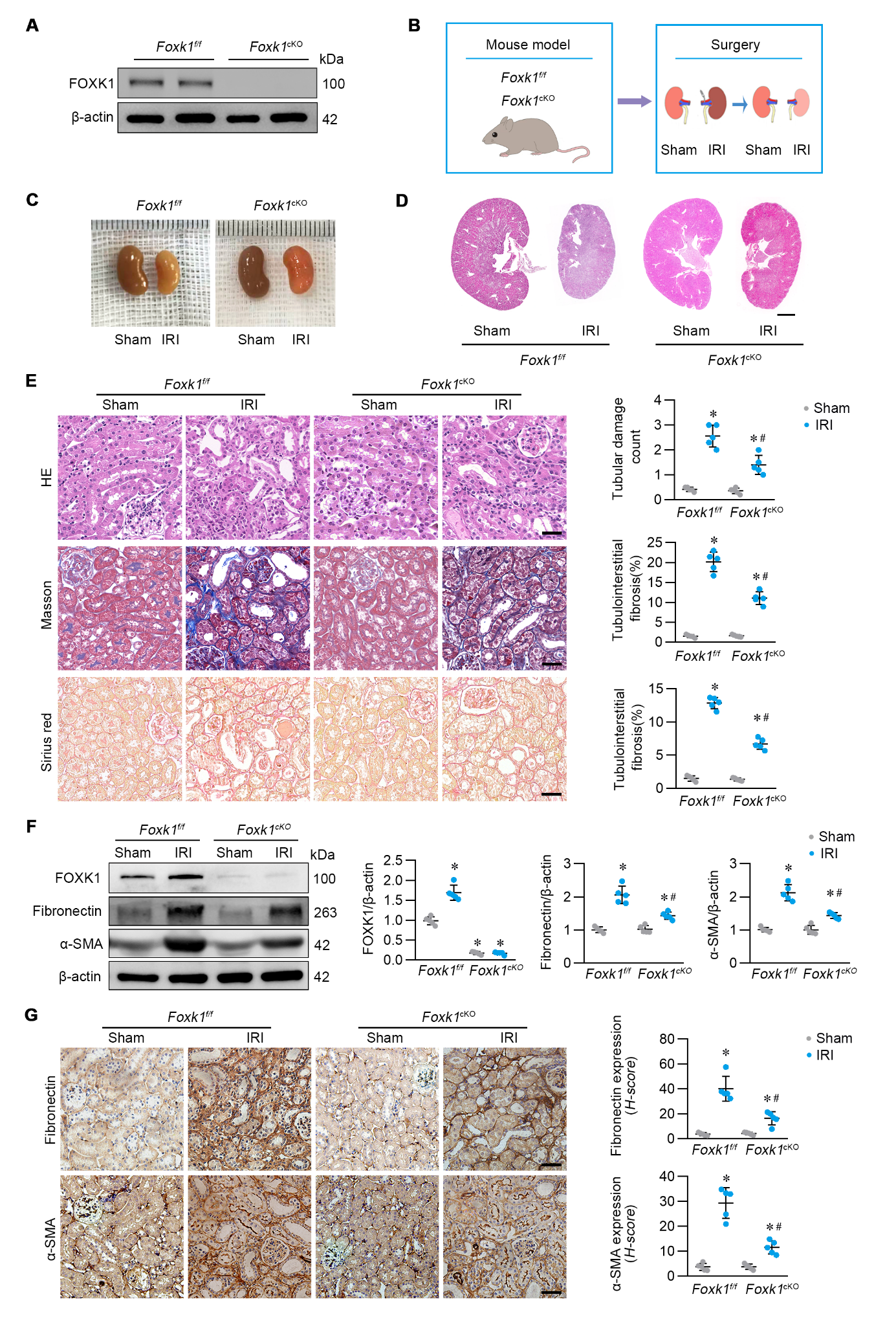


**Figure S2. Tubular-specific *Foxk1*-deficient mice ameliorate kidney fibrosis in IRI model.**

**A** Western blotting of FOXK1 expression in kidney tissues from *Foxk1^flox/flox^ and Foxk1^cKO^* mice. n=5 mice per group.

**B** Schematic diagram of generation of the IRI mouse model. Fourteen days after IRI, mice were sacrificed for kidney collection.

**C** Gross appearance of kidneys from the indicated groups.

**D** Photomicrographs exhibiting the Hematoxylin and eosin (H&E) staining of kidney sections from the indicated groups. Scale Bar=2 mm.

**E** H&E, Masson staining and Sirius red staining were applied to examine the tubular lesion and interstitial fibrosis in the renal tissue section from the indicated groups. Scale Bar=50 μm. n=5 mice per group.

**F** Western blotting of the protein expression of the related molecules in kidney tissues from the indicated group. n=5 mice per group. Quantitative data are expressed as the mean ± S.E.M. **P*< 0.05, compared to the sham group; *^#^P*< 0.05, compared with *Foxk1^flox/flox^* -IRI mice.

**G** Immunohistochemistry staining and quantifications of the protein expression of the related molecules in kidney tissues from the indicated group. Scale Bar=50 μm. n=5 mice per group. Quantitative data are expressed as the mean ± S.E.M. **P*< 0.05, compared to the sham group; *^#^P*< 0.05, compared with *Foxk1^flox/flox^* -IRI mice.


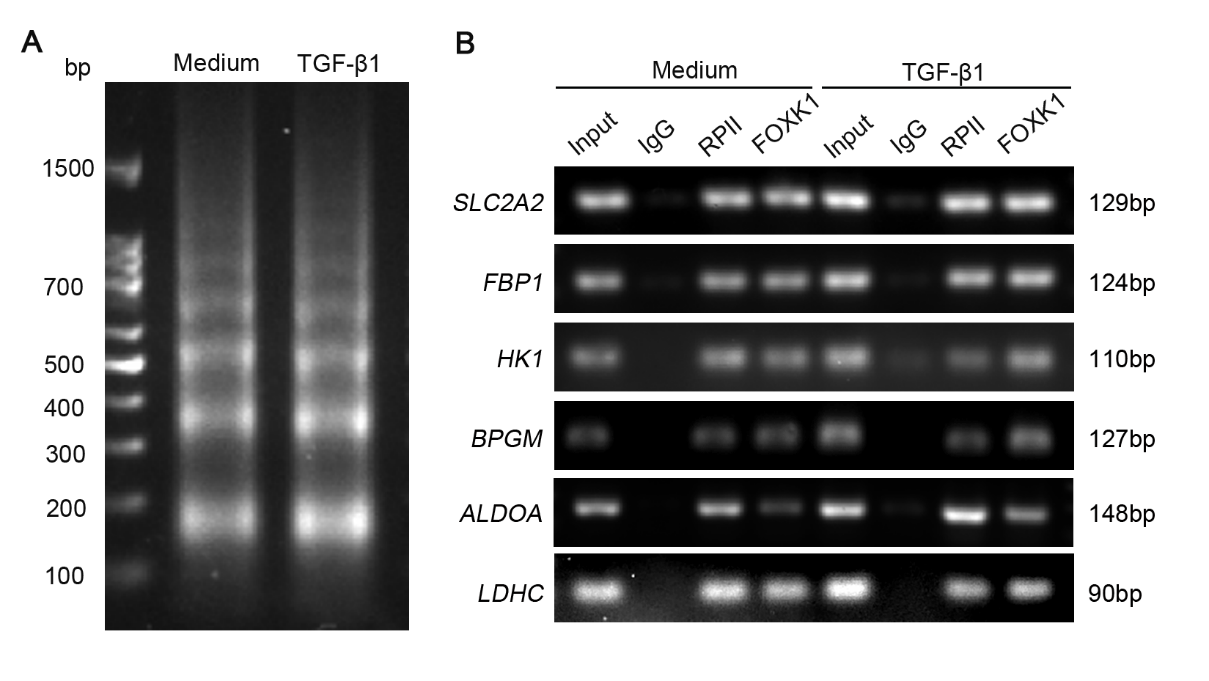


**Figure S3. ChIP-PCR detection of FOXK1.** Related to Figure 4.

**A** Agarose gel electrophoresis was performed to detect the effect of sonication of DNA samples.

**B** PCR amplification was carried out with DNA fragments that were immunoprecipitated by anti-FOXK1 (IP), anti-IgG (negative control) and anti-RNA POL II (positive control) and total DNA fragment (Input).


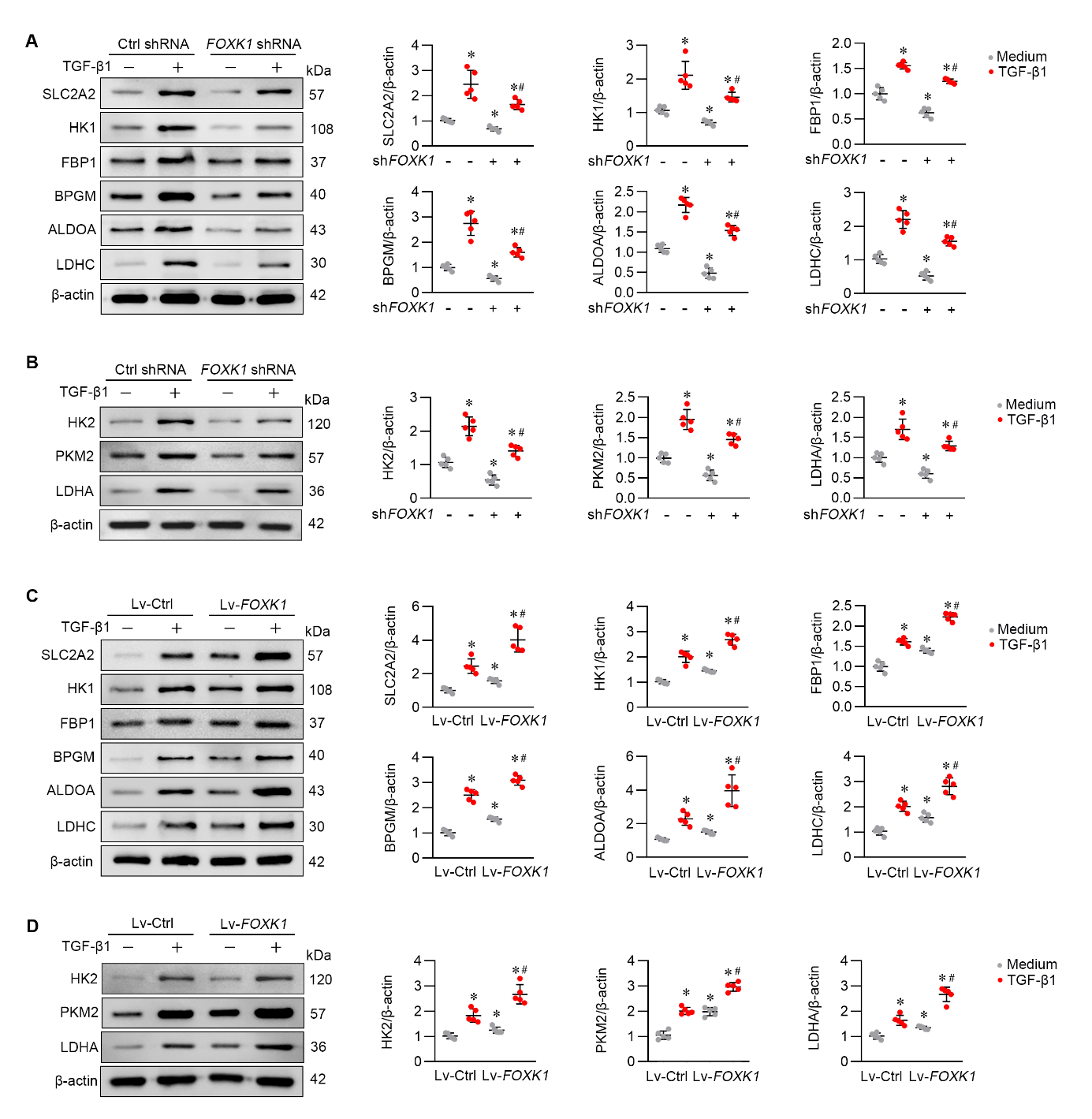


**Figure S4. FOXK1 enhances glycolysis related proteins expression in vitro.** Related to Figure 5.

**A, B** Western blots and quantitative results of SLC2A2, HK1, FBP1, BPGM, ALDOA, LDHC, HK2, PKM2, LDHA in human proximal tubular epithelial cell lines (HK-2) deleted FOXK1 with shRNA lentivirus for 24 h and treated with TGF-β1 (10 ng·ml^-1^) for 24 h. n = 5 samples per group.

**C, D** Western blots and quantitative results of SLC2A2, HK1, FBP1, BPGM, ALDOA, LDHC, HK2, PKM2, LDHA in HK-2 cells overexpressed FOXK1 with *FOXK1* lentivirus for 24 h and treated with TGF-β1 (10 ng·ml^-1^) for 24 h. n = 5 samples per group.


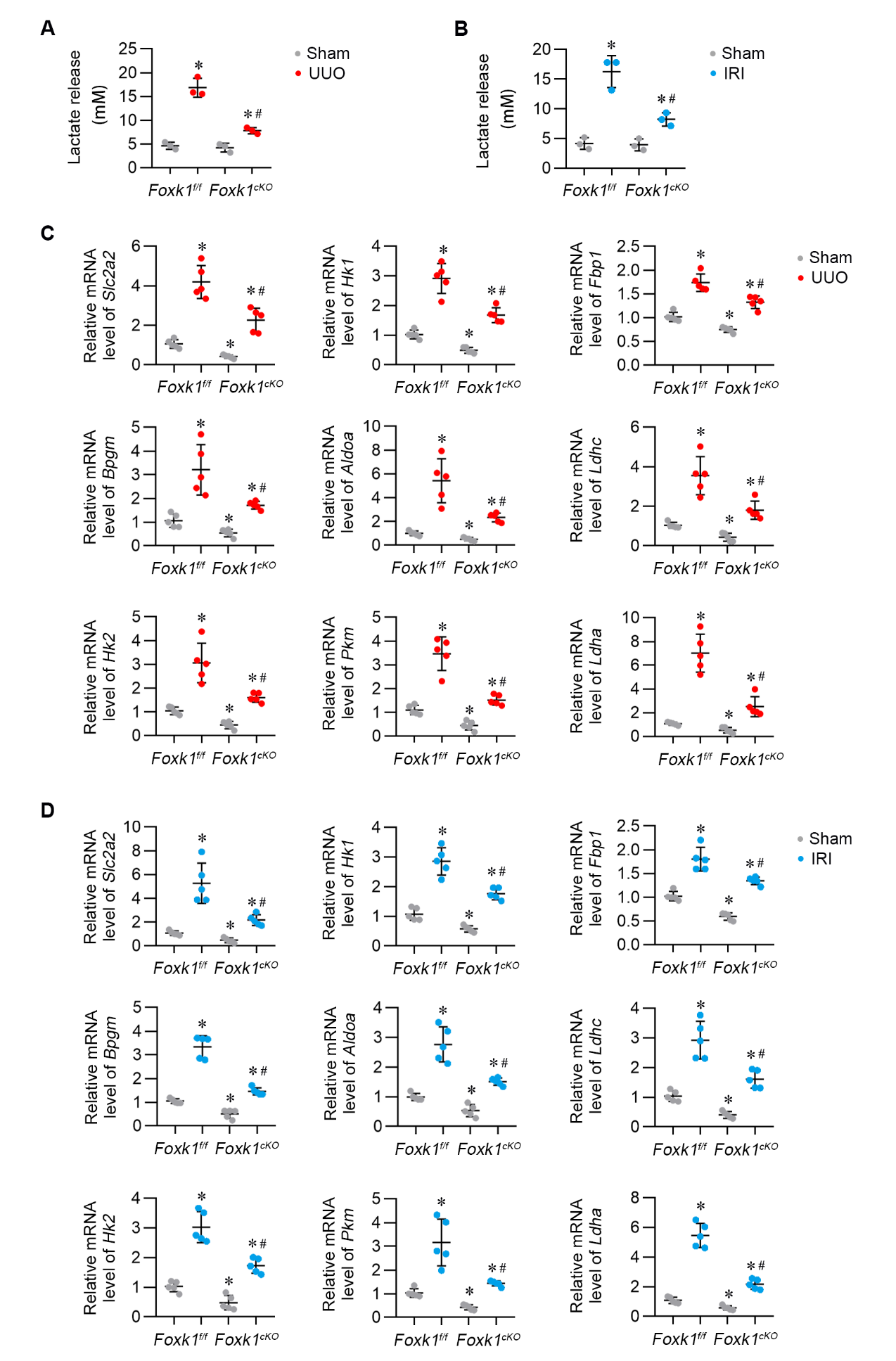


**Figure S5. The levels of glycolysis related genes and lactate in fibrotic mice kidney.** Related to Figure 5.

**A, B** Concentration of lactate was detected by ELISA kit from the indicated groups. (n = 3 mice per group).

**C, D** Relative mRNA levels of *Slc2a2, Hk1, Fbp1, Aldoa, Bpgm, Ldhc, Hk2, Pkm, Ldha* in the indicated groups (n = 5 mice per group).


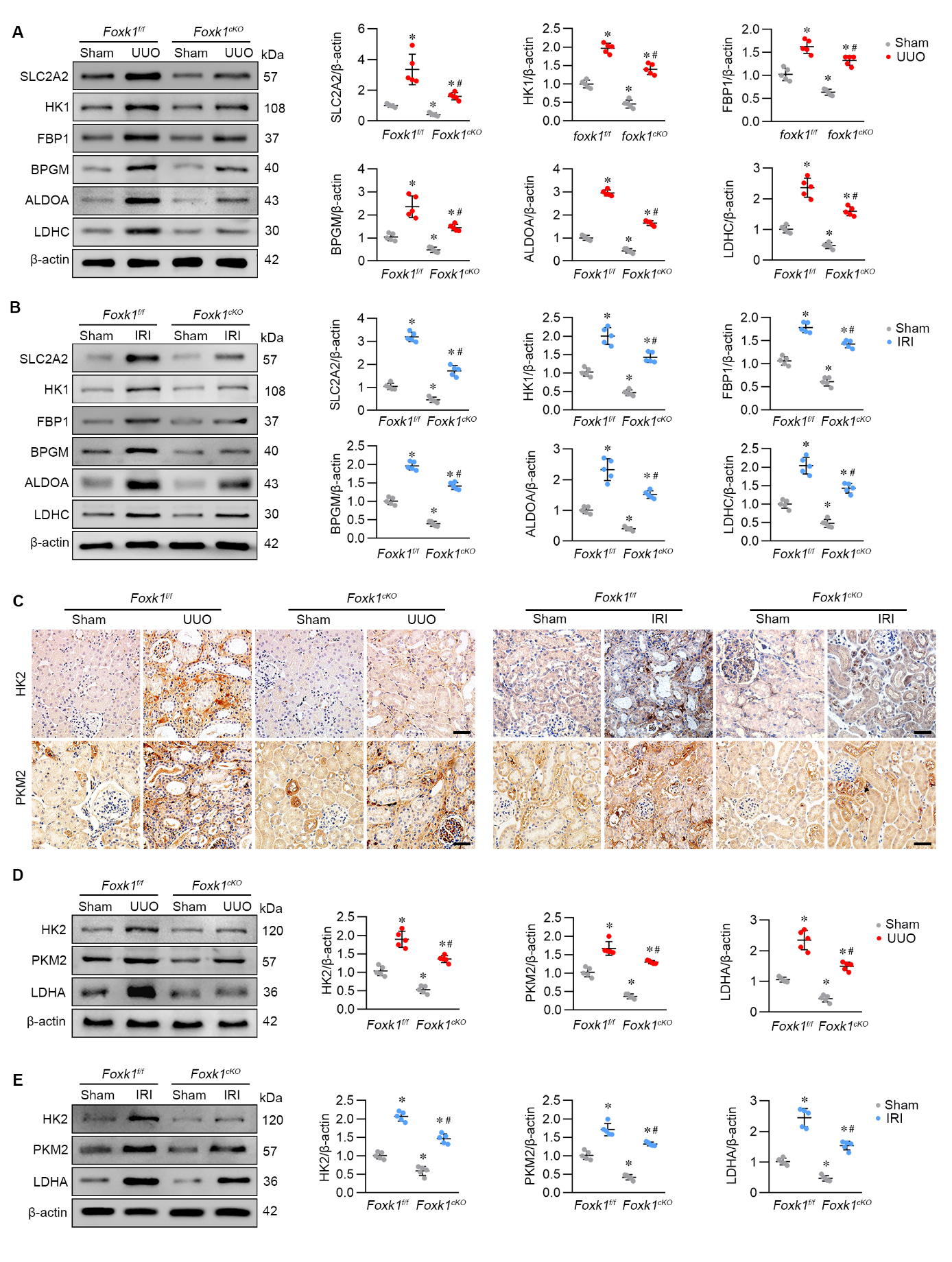


**Figure S6. FOXK1 enhances glycolysis related proteins expression in fibrotic mice kidney.** Related to Figure 5.

**A** Western blots and quantitative results of SLC2A2, HK1, FBP1, BPGM, ALDOA and LDHC in kidneys from *Foxk1* ^cKO^ mice and littermate controls after UUO. n = 5 mice per group.

**B** Western blots and quantitative results of HK2, PKM2 and LDHA in kidneys from *Foxk1* ^cKO^ mice and littermate controls after UUO. n = 5 mice per group.

**C** immunohistochemistry staining of HK2, PKM2 from *Foxk1* ^cKO^ mice and littermate controls after UUO. n = 5 mice per group. Scale bar= 50 μm.

**D, E** Western blots and quantitative results of SLC2A2, HK1, FBP1, BPGM, ALDOA, LDHC, HK2, PKM2 and LDHA in kidneys from *Foxk1* ^cKO^ mice and littermate controls after IRI. n = 5 mice per group.


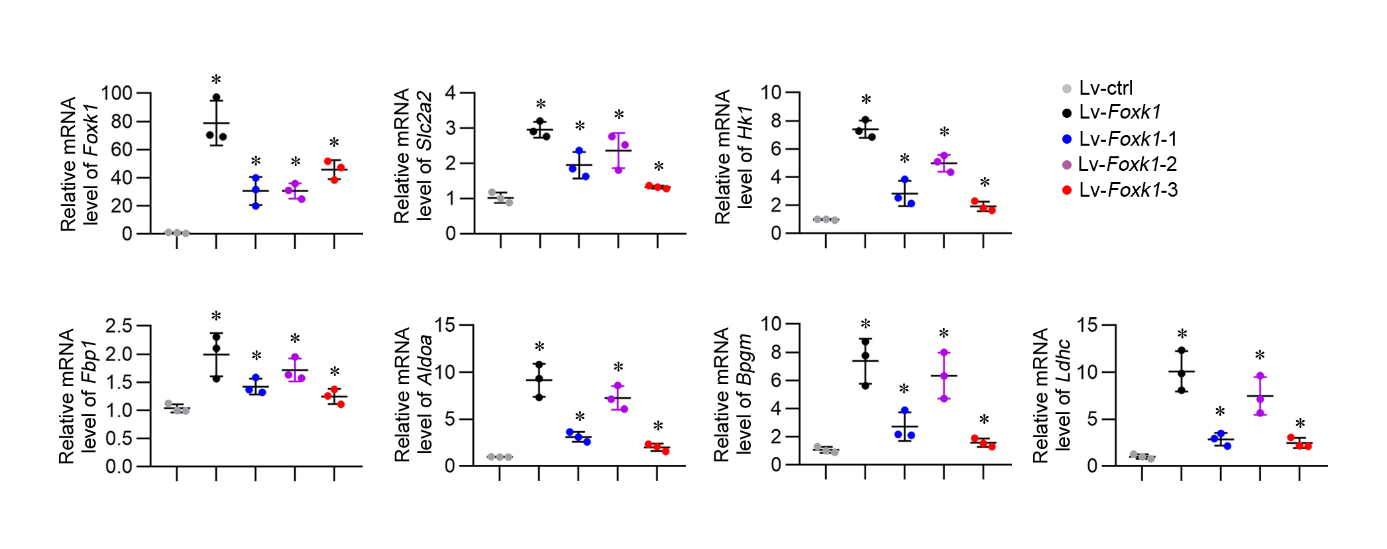


**Figure S7. FOXK1-mEGFP increased the mRNA levels of FOXK1 targeted glycolytic genes.** Related to Figure 7. TECs were isolated from *Foxk1* cKO mice and then transfected with Lv- *Foxk1*, Lv-*Foxk1*-1, Lv-*Foxk1*-2, Lv-*Foxk1*-3 and control lentivirus vectors. The relative mRNA level of *Foxk1, Slc2a2, Hk1, Fbp1, Aldoa, Bpgm, Ldhc* was detected. n = 3 biologically independent experiments. All data are represented as the mean value ± s.d. Significant differences were identified by student’s t-test. **P*< 0.05, compared to the control group.


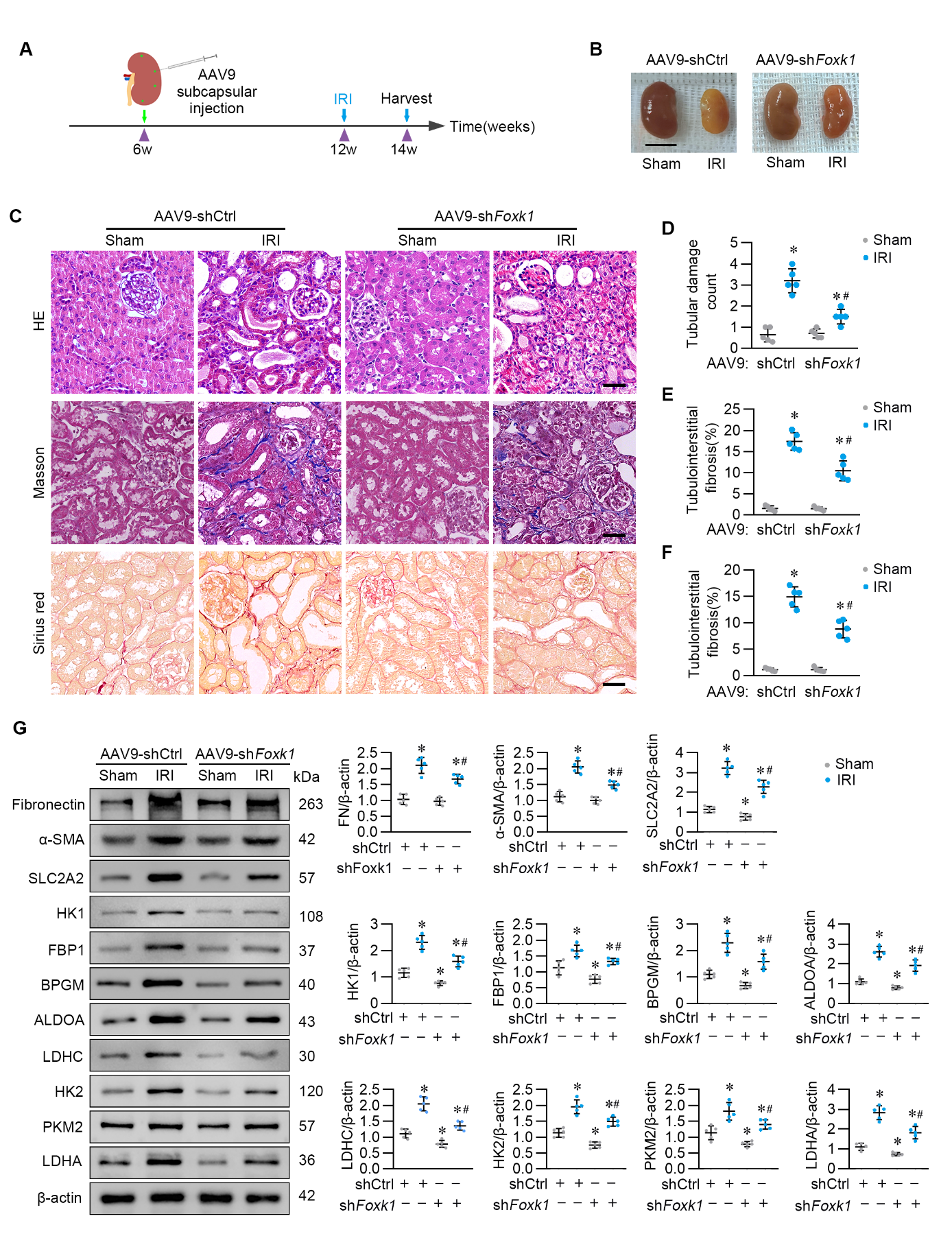


**Figure S8. AAV9-mediated knockdown of renal *Foxk1* ameliorate kidney fibrosis in IRI mice model.** Related to Figure 8.

**A** Schematic diagram of FOXK1 knockdown in mice. Renal subcapsular delivery of the AAV9-shControl or AAV9-sh*Foxk1* to wild-type C57BL/6 mice at six weeks of age. After the delivery for six weeks, the mice were subjected to IRI surgery.

**B** Gross appearance of kidneys from the indicated groups.

**C** H&E, Masson staining and Sirius red staining were applied to examine the tubular damage and tubulointerstitial fibrosis percentage in the renal tissue section from the indicated groups. Scale Bar= 50 μm. n=5 mice per group.

**D-F** Quantification of tubular damage score (**D**), and tubulointerstitial fibrosis percentage (**E,** **F**). n=5 mice per group. Quantitative data are expressed as the mean ± S.E.M. **P*< 0.05, compared to the sham group; *^#^P*< 0.05, compared with AAV9-shCtrl-IRI mice.

**G** Western blotting of the protein expression of the related molecules in kidney tissues from the indicated group. n=5 mice per group. Quantitative data are expressed as the mean ± S.E.M. **P*< 0.05, compared to the sham group; *^#^P*< 0.05, compared with AAV9-shCtrl-IRI mice.


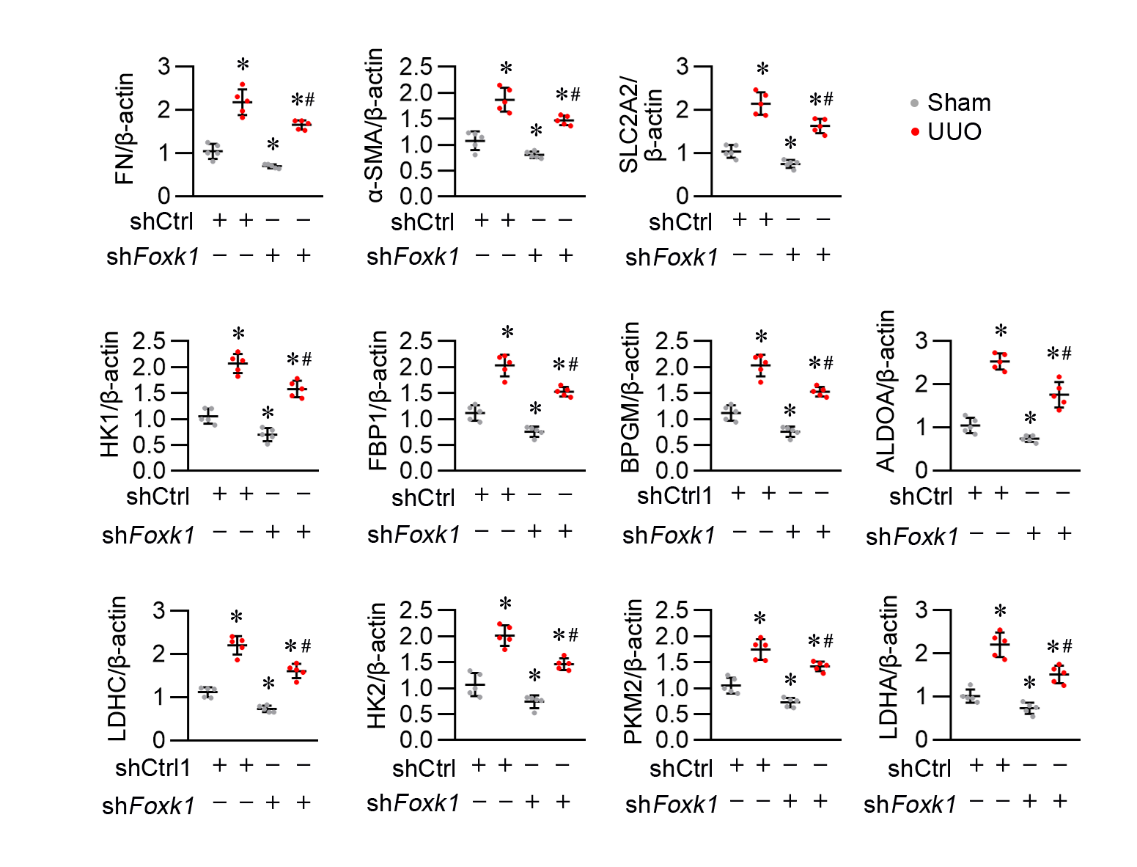


**Figure S9. AAV9-mediated knockdown of renal *Foxk1* downregulated kidney fibrotic markers in UUO mice model.** Related to Figure 8. Quantitative data are expressed as the mean ± S.E.M. n=5 mice per group. **P*< 0.05, compared to the sham group; *^#^P*< 0.05 compared with AAV9-shCtrl-UUO mice.

**Table S1.** **Baseline characteristics of CKD and adjacent normal tissues patients.**

| Sample number | Gender | Age [ys] | Serum  creatinine  [μmol/l] | Blood urea nitrogen [mmol/l] | eGFR | Interstitial  fibrosis (%) | Tubular damage score | Diagnosis | CKD stage |
| --- | --- | --- | --- | --- | --- | --- | --- | --- | --- |
| 1 | male | 61 | 98 | 8.21 | 71.01 | 3.094 | 0.5 | RCC | - |
| 2 | male | 56 | 112 | 10.58 | 82.67 | 5.748 | 1 | RCC | - |
| 3 | female | 56 | 106 | 9.48 | 85.71 | 2.296 | 0 | RCC | - |
| 4 | male | 70 | 112 | 7.35 | 81.07 | 7.889 | 1 | RCC | - |
| 5 | female | 65 | 130 | 8.19 | 75.97 | 3.208 | 0.5 | RCC | - |
| 6 | female | 67 | 113 | 8.54 | 68.7 | 1.754 | 0.5 | RCC | - |
| 7 | male | 32 | 66 | 5.65 | 105.74 | 1.523 | 0 | Normal kidney | - |
| 8 | female | 16 | 59 | 4.69 | 101.45 | 4.256 | 0 | Normal kidney | - |
| 9 | female | 29 | 65 | 6.13 | 117.76 | 21.999 | 5.5 | IgAN | CKD1 |
| 10 | male | 35 | 50 | 4.4 | 108.36 | 31.205 | 6.5 | MN | CKD1 |
| 11 | female | 36 | 62 | 4.73 | 106.04 | 57.289 | 2 | IgAN | CKD1 |
| 12 | female | 31 | 59 | 5.26 | 105 | 16.097 | 2.5 | FSGS | CKD1 |
| 13 | male | 42 | 45 | 7.37 | 104.86 | 39.573 | 5 | FSGS | CKD1 |
| 14 | male | 56 | 88 | 6.6 | 99.93 | 49.587 | 7 | MN | CKD1 |
| 15 | female | 31 | 86 | 10.8 | 89.88 | 13.42 | 5 | LN | CKD2 |
| 16 | female | 33 | 68 | 10.92 | 87.2 | 15.368 | 10 | MN | CKD2 |
| 17 | male | 27 | 74 | 6.38 | 76.21 | 4.73 | 9 | IgAN | CKD2 |
| 18 | female | 29 | 107 | 7.2 | 73.66 | 5.1 | 6 | IgAN | CKD2 |
| 19 | male | 30 | 93 | 7.26 | 72.29 | 34.367 | 4.5 | SGN | CKD2 |
| 20 | male | 45 | 79 | 10.11 | 67.47 | 24.251 | 3.5 | IgAN | CKD2 |
| 21 | male | 48 | 119 | 14.07 | 63.74 | 64.232 | 2 | DN | CKD2 |
| 22 | female | 56 | 123 | 6.97 | 58.6 | 45.324 | 1.5 | MN | CKD3 |
| 23 | female | 59 | 128 | 8.68 | 54.68 | 53.322 | 3.5 | DN | CKD3 |
| 24 | female | 64 | 139 | 8.01 | 50.78 | 13.244 | 7 | IgAN | CKD3 |
| 25 | female | 47 | 142 | 9.69 | 49.26 | 9.538 | 8.5 | SGN | CKD3 |
| 26 | female | 45 | 130 | 7.42 | 48.3 | 23.473 | 9 | IgAN | CKD3 |
| 27 | male | 61 | 140 | 8.61 | 45.74 | 41.245 | 12 | DN | CKD3 |
| 28 | female | 55 | 162 | 14.5 | 43.2 | 36.743 | 4 | MN | CKD3 |
| 29 | female | 39 | 129 | 5.46 | 42.79 | 46.244 | 7 | LN | CKD3 |
| 30 | female | 44 | 178 | 9.45 | 42.24 | 21.247 | 5.5 | IgAN | CKD3 |
| 31 | female | 61 | 58 | 5.83 | 41.1 | 20.458 | 6.5 | DN | CKD3 |
| 32 | female | 60 | 132 | 9.2 | 39.7 | 26.632 | 3.5 | MN | CKD3 |
| 33 | female | 55 | 128 | 8.71 | 39.11 | 31.003 | 9.5 | IgAN | CKD3 |
| 34 | male | 53 | 167 | 12.27 | 38.9 | 36.246 | 9.5 | IgAN | CKD3 |
| 35 | male | 44 | 173 | 15.04 | 37.77 | 9.031 | 10 | HRD | CKD3 |
| 36 | male | 42 | 192 | 10.4 | 32.2 | 12.083 | 6.5 | MN | CKD3 |
| 37 | female | 38 | 235 | 15.98 | 24.52 | 25.24 | 6.5 | DN | CKD4 |
| 38 | female | 51 | 204 | 24.8 | 22.98 | 26.321 | 3.5 | HRD | CKD4 |
| 39 | male | 46 | 287 | 10.46 | 21.8 | 40.023 | 3 | LN | CKD4 |
| 40 | female | 66 | 350 | 15.9 | 13.05 | 35.238 | 4.5 | LN | CKD5 |
| 41 | female | 69 | 727 | 16.04 | 8.04 | 27.201 | 4 | HRD | CKD5 |
| 42 | male | 54 | 518 | 11.55 | 7.06 | 24.101 | 7.5 | DN | CKD5 |
| 43 | male | 58 | 721 | 17.86 | 6.2 | 16.134 | 9 | SGN | CKD5 |
| 44 | female | 65 | 852 | 33.57 | 5.97 | 17.213 | 5.5 | SGN | CKD5 |
| 45 | female | 62 | 834 | 26.69 | 5.48 | 36.037 | 6.5 | LN | CKD5 |

IgAN: IgA nephropathy; MN: Membranous nephropathy; LN: lupus nephritis; FSGS: Focal segmental glomerular sclerosis; SGN: Sclerosing glomerulonephritis; DN: Diabetic Nephropathy; HRD: Hypertensive renal damage; RCC: Renal cell carcinoma. RCC: Renal cell carcinoma. eGFR [ml·min^-1^·(1.73m^2^) ^-1^]

**Table S2. Clinical information of renal obstruction patients.**

| Sample number | Gender |  | Age [years] | eGFR  [ml ·min^-1^ · (1.73m^2^)^-1^] | Diagnosis |
| --- | --- | --- | --- | --- | --- |
| 1 | female |  | 56 | 85.12 | Obstructive nephropathy |
| 2 | male |  | 52 | 90.56 | Obstructive nephropathy |
| 3 | female |  | 66 | 72.06 | Obstructive nephropathy |
| 4 | female |  | 63 | 95.16 | Obstructive nephropathy |
| 5 | male |  | 60 | 75.34 | Obstructive nephropathy |
| 6 | female |  | 62 | 83.69 | Obstructive nephropathy |

**Table S3. Genes enriched in the glycolysis/gluconeogenesis pathway related to Figure 4F.**

| **Gene ID** | **Gene symbol, description** |
| --- | --- |
| ENSG00000067057 | PFKP; ATP-Dependent 6-Phosphofructokinase, Platelet Type |
| ENSG00000067225 | PKM; pyruvate kinase |
| ENSG00000072210 | ALDH; Aldehyde Dehydrogenase 3 Family Member A2 |
| ENSG00000074800 | ENO1, eno; enolase 1 |
| ENSG00000079739 | PGM1; phosphoglucomutase 1 |
| ENSG00000091140 | DLD, lpd, pdhD; dihydrolipoamide dehydrogenase |
| ENSG00000100889 | PCK2; phosphoenolpyruvate carboxykinase (GTP) 6 |
| ENSG00000102144 | PGK1; phosphoglycerate kinase 1 |
| ENSG00000105220 | GPI; glucose-6-phosphate isomerase |
| ENSG00000105679 | GAPDHS; glyceraldehyde-3-phosphate dehydrogenase, spermatogenic |
| ENSG00000106633 | GCK; glucokinase |
| ENSG00000107789 | MINPP1; multiple inositol-polyphosphate phosphatase / 2,3-bisphosphoglycerate 3-phosphatase |
| ENSG00000108515 | ENO3; enolase 3 |
| ENSG00000108602 | ALDH3A1; Aldehyde Dehydrogenase 3 Family Member A1 |
| ENSG00000109107 | ALDOC; Aldolase, Fructose-Bisphosphate C |
| ENSG00000110435 | PDHX; dihydrolipoamide dehydrogenase-binding protein of pyruvate dehydrogenase complex; K13997 |
| ENSG00000111275 | ALDH2; Aldehyde Dehydrogenase 2 Family Member |
| ENSG00000111640 | GAPDH, gapA; glyceraldehyde 3-phosphate dehydrogenase |
| ENSG00000111669 | TPI1; triosephosphate isomerase 1 |
| ENSG00000111674 | ENO2; enolase 2 |
| ENSG00000111716 | LDHB; Lactate Dehydrogenase B |
| ENSG00000117448 | AKR1A1; Aldo-Keto Reductase Family 1 Member A1 |
| ENSG00000124253 | PCK1; phosphoenolpyruvate carboxykinase 1 |
| ENSG00000130957 | FBP2; Fructose-Bisphosphatase 2 |
| ENSG00000131069 | ACSS1_2, acs; acetyl-CoA synthetase |
| ENSG00000131482 | G6PC; glucose-6-phosphatase |
| ENSG00000131828 | PDHA1; pyruvate dehydrogenase E1 component alpha subunit |
| ENSG00000132746 | ALDH3B2; Aldehyde Dehydrogenase 3 Family Member B2 |
| ENSG00000134333 | LDHA; Lactate Dehydrogenase A |
| ENSG00000136872 | ALDOB; Aldolase, Fructose-Bisphosphate B |
| ENSG00000137124 | ALDH1B1; Aldehyde Dehydrogenase 1 Family Member B1 |
| ENSG00000141349 | G6PC3; lucose-6-Phosphatase Catalytic Subunit 3 |
| ENSG00000141959 | PFKL; Phosphofructokinase, Liver Tye |
| ENSG00000143149 | ALDH9A1; aldehyde dehydrogenase family 9 member A1 |
| ENSG00000143627 | PKLR; pyruvate kinase isozymes R/L |
| ENSG00000143891 | GALM;Galactose Mutarotase |
| ENSG00000149925 | ALDOA; Aldolase, Fructose-Bisphosphate A |
| ENSG00000152254 | G6PC2; Glucose-6-Phosphatase Catalytic Subunit 2 |
| ENSG00000154930 | ACSS1_2, acs; acetyl-CoA synthetase |
| ENSG00000156510 | HKDC1; Hexokinase Domain-Containing Protein 1 |
| ENSG00000156515 | HK1; hexokinase 1 |
| ENSG00000159322 | ADPGK; ADP-dependent glucokinase |
| ENSG00000159399 | HK2; hexokinase 2 |
| ENSG00000160883 | HK3; hexokinase 3 |
| ENSG00000163114 | PDHA2; Pyruvate Dehydrogenase E1 Subunit Alpha 2 |
| ENSG00000164708 | PGAM2; Phosphoglycerate Mutase 2 |
| ENSG00000164904 | ALDH7A1; aldehyde dehydrogenase family 7 member A1 |
| ENSG00000165140 | FBP1; Fructose-Bisphosphatase 1 |
| ENSG00000166796 | LDHC; L-lactate dehydrogenase C |
| ENSG00000166800 | LDHAL6A; Lactate Dehydrogenase A Like 6A |
| ENSG00000168291 | PDHB, pdhB; pyruvate dehydrogenase E1 component beta subunit |
| ENSG00000169299 | PGM2; phosphoglucomutase / phosphopentomutase |
| ENSG00000170950 | PGK2; phosphoglycerate kinase 2 |
| ENSG00000171989 | LDHAL6B; Lactate Dehydrogenase A Like 6B |
| ENSG00000172331 | BPGM; bisphosphoglycerate/phosphoglycerate mutase |
| ENSG00000172955 | ADH6; alcohol dehydrogenase 6 |
| ENSG00000187758 | ADH1_7; alcohol dehydrogenase 1/7 |
| ENSG00000117394 | SLC2A1, GLUT-1; Solute Carrier Family 2 Member 1 |
| ENSG00000198099 | ADH4; alcohol dehydrogenase 4 |
| ENSG00000226784 | PGAM, gpmA; 2,3-bisphosphoglycerate-dependent phosphoglycerate mutase |
| ENSG00000158571 | PFKFB1; 6-phosphofructo-2-kinase / fructose-2,6-biphosphatase 1 |
| ENSG00000278373 | G6PC; glucose-6-phosphatase |
| ENSG00000282019 | GPI, pgi; glucose-6-phosphate isomerase |
| ENSG00000067057 | PFKP; ATP-Dependent 6-Phosphofructokinase, Platelet Type |
| ENSG00000067225 | PKM; pyruvate kinase |
| ENSG00000072210 | ALDH; Aldehyde Dehydrogenase 3 Family Member A2 |
| ENSG00000074800 | ENO1, eno; enolase 1 |
| ENSG00000079739 | PGM1; phosphoglucomutase 1 |
| ENSG00000091140 | DLD, lpd, pdhD; dihydrolipoamide dehydrogenase |
| ENSG00000100889 | PCK2; phosphoenolpyruvate carboxykinase (GTP) 6 |
| ENSG00000102144 | PGK1; phosphoglycerate kinase 1 |
| ENSG00000105220 | GPI; glucose-6-phosphate isomerase |
| ENSG00000105679 | GAPDHS; glyceraldehyde-3-phosphate dehydrogenase, spermatogenic |
| ENSG00000106633 | GCK; glucokinase |

**Table S4. Resources list.**

| **REAGENT or RESOURCE** | **SOURCE** | **IDENTIFIER** |
| --- | --- | --- |
| **1. Antibodies** | | |
| FOXK1 | Abcam | Cat#ab18196 |
| Fibronectin | Abcam | Cat#ab45688 |
| α-SMA | Abcam | Cat#ab7817 |
| SLC2A2 | Abclonal | Cat#A12307 |
| FBP1 | Abclonal | Cat#A5406 |
| BPGM | Abclonal | Cat#A7880 |
| ALDOA | Abclonal | Cat#A1142 |
| LDHA | Abclonal | Cat#A21893 |
| LDHC | Abclonal | Cat#A15003 |
| PKM2 | Cell Signaling Technology (CST) | Cat#D78A4 |
| HK2 | Santa Cruz | Cat#sc-374091 |
| HK1 | Servicebio | Cat#GB11657 |
| β-actin | Servicebio | Cat#GB15001 |
| RNA Pol II | EPIGENTEK | Cat#A-2033 |
| POLR2K | GeneTex | Cat#GTX132871 |
| Alexa Fluor 488- and 594-  conjugated secondary antibodies | Thermo Fisher | Cat#A-11008 &  Cat#A-11012 |
| Horseradish peroxidase (HRP)-  coupled goat anti-mouse/rabbit IgG | Thermo Fisher | Cat#A16066 &  Cat#65-6120 |
| Fluorescein-labeled lotus tetragonolobus lectin (LTL) | Vector | FL-1321 |
| Fluorescein-dolichos biflorus agglutinin (DBA) | Vector | FL-1031 |
| **2. Bacterial and virus strains** | | |
| FOXK1 silenced lentivirus expressing short hairpin RNA (shRNA) | Genechem Co. | N/A |
| FOXK1 overexpressed lentivirus expressing short hairpin RNA (shRNA) | Huameng Biotechnology | N/A |
| FOXK1 overexpressed lentivirus expressing short hairpin RNA (shRNA) targeting the truncated sequence | Huameng Biotechnology | N/A |
| **3. Biological samples** | | |
| Human kidney tissues from patients with CKD | Renmin hospital of Wuhan University | N/A |
| Human adjacent normal tissues | Renmin hospital of Wuhan University | N/A |
| Demographic and clinical information of patients see Table S1-S2 (Supporting Information). | Renmin hospital of Wuhan University | N/A |
| **4. Chemicals, peptides, and recombinant proteins** | | |
| Recombinant human TGF-β1 | MCE | Cat#HY-P7118 |
| FOXK1-mEGFP fusion proteins | Sino Biological | N/A |
| Fetal bovine serum (FBS) | Gibco | Cat#10099-141 |
| DMEM-F12 | Hyclone | Cat#SH30023.01 |
| penicillin/streptomycin | Hyclone | Cat#SV30010 |
| DAPI | Sigma | Cat#S7113 |
| Tryptic digestion solution | Beyotime | Cat#C0203 |
| **5. Commercial assays** | | |
| Seahorse XF Glycolytic Rate Assay Kit | Seahorse Bioscience | Cat#103344-100 |
| Seahorse XF Cell Mito Stress Test Kit | Seahorse Bioscience | Cat#103015-100 |
| Pierce Magnetic ChIP kit | Thermo scientific | Cat#26157 |
| One Step Mouse Genotyping Kit | Vazyme | Cat#PD101-01 |
| Lactate Assay Kit | Cayman | Cat#700510 |
| Lactate Assay Kit | Jiancheng Bio. | Cat#A019-2-1 |
| **6. Experimental models: Cell lines** | | |
| HK-2 (human kidney 2) cell line | ATCC | Cat#CBP60447 |
| Mouse proximal tubular (BUMPT) cell line |  |  |
| **7. Experimental models: Strains** | | |
| *Ggt1-Cre* mice | Cyagen | Cat#C001028 |
| *Foxk1 ^flox/flox^* | Cyagen | N/A |
| **8. Oligonucleotides** | | |
| Primers for RT-qPCR, see Table S5(Supporting Information). | This paper | N/A |
| shRNA sequences for *Foxk1*: 5′-CUCUCUUUGAACCGUUACUTT-3′ | This paper | N/A |

**Table S5. Primer sequences used in this study.**

| Target | Species | Primer sequences (5’-3’) | Amplicon size(bp) |
| --- | --- | --- | --- |
| *LDHA* | Human | Forward ATGGCAACTCTAAAGGATCAGC | 86 |
|  |  | Reverse CCAACCCCAACAACTGTAATCT |  |
| *LDHB* | Human | Forward CCTCAGATCGTCAAGTACAGTCC | 113 |
|  |  | Reverse ATCACGCGGTGTTTGGGTAAT |  |
| *LDHC* | Human | Forward AGAACATGGTGATTCTAGTGTGC | 191 |
|  |  | Reverse ACAGTCCAATAGCCCAAGAGG |  |
| *LDHD* | Human | Forward CCGTAGCCCGCATTGAGTT | 161 |
|  |  | Reverse CTGCTGGACTATCTCCTCTGT |  |
| *SLC2A1* | Human | Forward TCTGGCATCAACGCTGTCTTC | 94 |
|  |  | Reverse CGATACCGGAGCCAATGGT |  |
| *SLC2A2* | Human | Forward GGGCAATTATGATCTGTGGCA | 228 |
|  |  | Reverse TTCTGCTCACTCGATGCTTCT |  |
| *SLC2A3* | Human | Forward GCTGGGCATCGTTGTTGGA | 123 |
|  |  | Reverse GCACTTTGTAGGATAGCAGGAAG |  |
| *SLC2A4* | Human | Forward TGGGCGGCATGATTTCCTC | 88 |
|  |  | Reverse GCCAGGACATTGTTGACCAG |  |
| *HK1* | Human | Forward GCTCTCCGATGAAACTCTCATAG | 121 |
|  |  | Reverse GGACCTTACGAATGTTGGCAA |  |
| *HK2* | Human | Forward GAGCCACCACTCACCCTACT | 249 |
|  |  | Reverse CCAGGCATTCGGCAATGTG |  |
| *HK3* | Human | Forward GGACAGGAGCACCCTCATTTC | 97 |
|  |  | Reverse CCTCCGAATGGCATCTCTCAG |  |
| *HK4* | Human | Forward GCAGAAGGGAACAATGTCGTG | 121 |
|  |  | Reverse CGTAGTAGCAGGAGATCATCGT |  |
| *HKDC1* | Human | Forward TGAGCCGTCTGACCAAAGC | 112 |
|  |  | Reverse TAGGGGTCGTCATAGGCACA |  |
| *PKM* | Human | Forward ATGTCGAAGCCCCATAGTGAA | 118 |
|  |  | Reverse TGGGTGGTGAATCAATGTCCA |  |
| *ALDOA* | Human | Forward GCTGTCACTGGGATCACCTTC | 249 |
|  |  | Reverse GCTCGGAGTGTACTTTCCTTGA |  |
| *ALDOB* | Human | Forward TGTCTGGTGGCATGAGTGAAG | 104 |
|  |  | Reverse GGCCCGTCCATAAGAGAAACTT |  |
| *ALDOC* | Human | Forward ATGCCTCACTCGTACCCAG | 151 |
|  |  | Reverse TTTCCACCCCAATTTGGCTCA |  |
| *PFKFB1* | Human | Forward TACCAGAGAACGACGGTCACT | 101 |
|  |  | Reverse CTGCAATTATGCCAGGGTCATTA |  |
| *PFKFB2* | Human | Forward TGATGCCACCAATACAACCCG | 95 |
|  |  | Reverse CACAGACGGATTCCACAAAGA |  |
| *PFKFB3* | Human | Forward AGCCCGGATTACAAAGACTGC | 82 |
|  |  | Reverse GGTAGCTGGCTTCATAGCAAC |  |
| *PFKFB4* | Human | Forward CAACATCGTGCAAGTGAAACTG | 111 |
|  |  | Reverse GACTCGTAGGAGTTCTCATAGCA |  |
| *PFKP* | Human | Forward GACCTTCGTTCTGGAGGTGAT | 152 |
|  |  | Reverse CACGGTTCTCCGAGAGTTTG |  |
|  | Human | Forward CAGTTACCGCTTTGTGCAGAA | 127 |
| *FOXK1* |  | Reverse CGGCTTTGACTCATCCTTGG |  |
|  | Mouse | Forward ACCCACGAATAGCTTGACTGG | 156 |
|  |  | Reverse GCATTAGCGGCTACTGAGACG |  |
|  | Human | Forward CGGTGGCTGTCAGTCAAAG | 130 |
| *FN1* |  | Reverse AAACCTCGGCTTCCTCCATAA |  |
|  | Mouse | Forward ATGTGGACCCCTCCTGATAGT | 124 |
|  |  | Reverse GCCCAGTGATTTCAGCAAAGG |  |
|  | Human | Forward AAAAGACAGCTACGTGGGTGA | 76 |
| *ACTA2* |  | Reverse GCCATGTTCTATCGGGTACTTC |  |
| *(α-SMA)* | Mouse | Forward GTCCCAGACATCAGGGAGTAA | 102 |
|  |  | Reverse TCGGATACTTCAGCGTCAGGA |  |
